# Ubiquitin-Dependent Modification of Skeletal Muscle by the Parasitic Nematode, *Trichinella spiralis*

**DOI:** 10.1371/journal.ppat.1005977

**Published:** 2016-11-21

**Authors:** Rhiannon R. White, Amy H. Ponsford, Michael P. Weekes, Rachel B. Rodrigues, David B. Ascher, Marco Mol, Murray E. Selkirk, Steven P. Gygi, Christopher M. Sanderson, Katerina Artavanis-Tsakonas

**Affiliations:** 1 Department of Life Sciences, Imperial College London, London, United Kingdom; 2 Department of Cellular and Molecular Physiology, Institute of Translational Medicine, University of Liverpool, Liverpool, United Kingdom; 3 Cambridge Institute for Medical Research, University of Cambridge, Cambridge, United Kingdom; 4 Department of Cell Biology, Harvard Medical School, Boston, United States of America; 5 Department of Biochemistry, University of Cambridge, Cambridge, United Kingdom; 6 Department of Biochemistry, University of Melbourne, Melbourne, Australia; 7 Department of Pathology, University of Cambridge, Cambridge, United Kingdom; University of Medicine & Dentistry of New Jersey, UNITED STATES

## Abstract

*Trichinella spiralis* is a muscle-specific parasitic worm that is uniquely intracellular. *T*. *spiralis* reprograms terminally differentiated skeletal muscle cells causing them to de-differentiate and re-enter the cell cycle, a process that cannot occur naturally in mammalian skeletal muscle cells, but one that holds great therapeutic potential. Although the host ubiquitin pathway is a common target for viruses and bacteria during infection, its role in parasite pathogenesis has been largely overlooked. Here we demonstrate that the secreted proteins of *T*. *spiralis* contain E2 Ub-conjugating and E3 Ub-ligase activity. The E2 activity is attributed to *Ts*UBE2L3, a novel and conserved *T*. *spiralis* enzyme located in the secretory organ of the parasite during the muscle stages of infection. *Ts*UBE2L3 cannot function with any *T*.*spiralis* secreted E3, but specifically binds to a panel of human RING E3 ligases, including the RBR E3 ARIH2 with which it interacts with a higher affinity than the mammalian ortholog UbcH7/UBE2L3. Expression of *Ts*UBE2L3 in skeletal muscle cells causes a global downregulation in protein ubiquitination, most predominantly affecting motor, sarcomeric and extracellular matrix proteins, thus mediating their stabilization with regards to proteasomal degradation. This effect is not observed in the presence of the mammalian ortholog, suggesting functional divergence in the evolution of the parasite protein. These findings demonstrate the first example of host-parasite interactions via a parasite-derived Ub conjugating enzyme; an E2 that demonstrates a novel muscle protein stabilization function.

## Author Summary

Parasitic worms often establish long-lasting infections in their hosts; tightly regulating their surroundings to strike a delicate balance between host cell modulation and protection that will ensure their replication. This is accomplished via the active secretion of parasite glycolipids and glycoproteins into the host. *Trichinella spiralis*, a parasitic nematode that infects skeletal muscle of mammals, birds and reptiles, is the only parasitic worm with a true intracellular stage. *T*. *spiralis* invade terminally differentiated myotubes, reprogramming them to de-differentiate and re-enter the cell cycle, a process that cannot occur naturally in mammalian skeletal muscle cells, but one that holds great therapeutic potential. We have identified and characterized a novel *T*. *spiralis* secreted protein that, despite a high level of sequence identity, appears to have evolved a different function to its host ortholog. This protein is an active Ub conjugating enzyme that binds to a panel of human E3 Ub ligases with higher affinity than the host ortholog. Furthermore, when expressed in skeletal muscle cells in culture, its presence uniquely leads to the stabilization of muscle-specific proteins via the downregulation of their ubiquitination.

## Introduction

The ubiquitin (Ub) pathway is essential for post-translational protein regulation in eukaryotic cells, controlling many important cellular processes such as transcription, cell cycle, differentiation and apoptosis ([1,2]. Ub is a 76 amino acid protein that, in a highly regulated fashion, is covalently conjugated to substrate proteins via an E1 activating, E2 conjugating and E3 ligating enzyme cascade[3]. Ubiquitination regulates the fate and function of the substrate, to maintain a healthy homeostasis within the cell. The particular outcome is determined by a combination of possible variables, for example the specific lysine residue attachment site on the protein, the length and type of Ub chain, and the number of total Ub moieties on a single protein[4,5]. Since the specific E2:E3 enzyme pair denotes substrate and moiety specificity, ubiquitination is highly regulated by the abundance, localization and activity of these Ub-specific enzymes.

Considering the important role of the Ub pathway in maintaining the healthy homeostasis of a cell, and therefore the healthy physiology of an organism, it is not surprising that its disruption is directly implicated in infection and disease. Prokaryotes do not have an endogenous Ub pathway, however certain viral and bacterial pathogens encode Ub-specific enzymes that target host Ub machinery for enhanced virulence and immune evasion[6–9]. Although much attention has been aimed at understanding the role of the Ub pathway in infection by pathogenic viruses and bacteria, very little is known about how parasites may interfere with host ubiquitination. Parasites are eukaryotic and therefore already express endogenous Ub machinery. To date, there are only two reports of the direct targeting of the host Ub pathway during parasitic infection. The first involves indirect communication with the host Ub system by the *Toxoplasma* dense granule protein GRA16. GRA16 is exported into the host cell nucleus and binds the host Ub hydrolase HAUSP, modulating the cell cycle via HAUSP-dependent p53 regulation[10,11]. The second involves direct communication with the host Ub pathway by a *Trypanosoma cruzi* active RING domain secretory protein SPRING. Hashimoto *et al* showed that *in vitro*, SPRING is able to catalyze Ub conjugation with human UbcH5 and 13.

*Trichinella spiralis* is a promiscuous parasitic nematode that infects skeletal muscle cells of mammals, birds and reptiles. The parasite is propagated by the consumption of infected tissue and undergoes the same life cycle stages irrespective of the host. The longest life cycle stage is intracellular, giving the parasite direct access to host intracellular machinery. The severity of the associated disease, trichinellosis, depends on the infection load and the presentation varies in severity correspondingly from asymptomatic to fatal. During the chronic intracellular phase of infection, *T*. *spiralis* invade terminally differentiated (TD) myotubes, releasing a mixture of secreted products (SP) including glycoproteins and glycolipids into the cytoplasm and nucleus of the host cell. The host cell undergoes a dramatic program of de-differentiation and cell cycle re-entry followed by cell cycle arrest, initiated and characterized by a change in transcriptional profile, a downregulation of host muscle specific proteins such as myogenin and myosin heavy chain and the loss of identity and function as a myotube[12,13]. This coincides with a change in morphology, as the cell is transformed from a long, linear fiber into a fat, oval-shaped structure termed a nurse cell ([14–16]. The parasite resides inside the nurse cell until a chance transmission occurs. It is thought that this process of nurse cell development is induced by the *T*. *spiralis* SP via direct communication with host cell proteins and genetic material.

In this study we used muscle-stage *T*. *spiralis* as a model to investigate whether eukaryotic parasites have evolved strategies to target the Ub pathway during infection. Not only does *T*. *spiralis* SP contain both E2 Ub conjugating and E3 Ub ligase activity, we were able to attribute the E2 activity to the secretion of *Ts*UBE2L3, an enzyme that is located in the secretory organ of *T*. *spiralis* during infection. We show that *Ts*UBE2L3 interacts with the host E3 ARIH2 with higher binding affinity than the endogenous mammalian ortholog. Furthermore, *Ts*UBE2L3 causes a significant downregulation in the levels of ubiquitination of motor, sarcomeric and extracellular matrix proteins. These findings demonstrate the first example of host-parasite interaction via a parasite-derived Ub conjugating enzyme that can stabilize skeletal muscle-specific proteins by inhibiting their ubiquitination.

## Results

### Intracellular-stage *T*. *spiralis* secreted products (SP) demonstrate Ub conjugation and ligation activities

**Fig 1:**
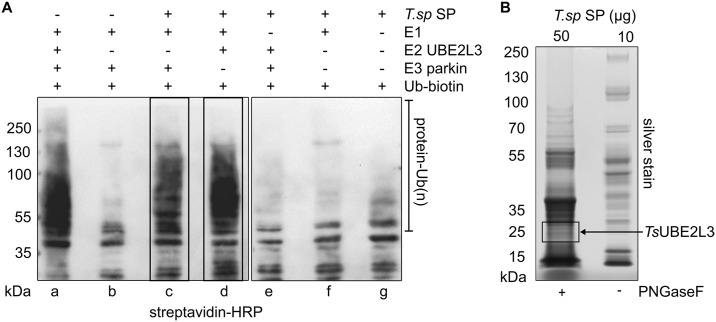
Identification of E2 and E3 activity in *T*. *spiralis* SP. **A.** Streptavidin-HRP blot of *in vitro* ubiquitination reactions including 1) human E1 (UBE1A), E2 (UBE2L3), E3 (parkin), and Ub-biotin, 2) in the absence of the E2, 3) SP substituted for the human E2, 4) SP substituted for the human E3, 5) SP substituted for the human E1, 6) SP substituted for both the human E2 and E3, 7) SP and Ub-biotin alone. **B.** *T*.*sp* SP were separated by SDS-PAGE and visualized by silver-staining. 50 μg of SP were de-glycosylated by PNGase treatment and analyzed by LC/MS/MS (10 μg of glycosylated proteins were analyzed for comparison). Peptides matching a *T*. *spiralis* E2, *Ts*UBE2L3, were identified from the boxed section of the gel.

An *in vitro* Ub conjugation assay was carried out to determine if the secreted products (SP) of *T*. *spiralis* muscle larvae contain E1, E2 or E3 enzymatic activity. All reactions were separated by SDS-PAGE and analyzed by streptavidin blot (Fig 1A). As a positive control, a reaction containing human recombinant E1 (*Hs*UBE1A), human recombinant E2 (*Hs*UBE2L3), and human recombinant E3 (parkin) was analyzed. In the presence of *Hs*UBE2L3, parkin (51 kDa) was able to auto-ubiquitinate, visible as a streptavidin-reactive smear (Fig 1A). When *Hs*UBE2L3 was removed from the reaction no signal was observed (b), confirming the requirement of the E2 for parkin auto-ubiquitination. *T*. *spiralis* SP were then substituted into the reaction for either the E2, E3 or E1. A reaction containing *T*. *spiralis* SP plus *Hs*UBE1A only was also analyzed, as well as a reaction containing only *T*. *spiralis* SP and biotin-Ub as a negative control. A streptavidin-reactive smear, representing either parkin-Ub or SP-Ub, was observed when *T*. *spiralis* SP were substituted in the place of *Hs*UBE2L3. Signal representing SP-Ub was also observed when *T*. *spiralis* SP were substituted in the place of parkin. No ubiquitination was observed when *T*. *spiralis* SP were substituted in the place of *Hs*UBE1A, or when the E1 and SP were reacted alone. This suggests that proteins in *T*. *spiralis* SP have E2 and E3 but not E1 activities. Since these proteins are activated only by the addition of the human E1 plus E2, or the human E1 plus E3, it is unlikely that the *T*. *spiralis* secreted E2 and E3 can work together without an external enzyme source. It was therefore hypothesized that ubiquitination of substrates by *T*. *spiralis* muscle larvae SP requires mammalian host E2 or E3 partners.

### Proteomic analysis of *T*. *spiralis* SP identifies a Ub conjugating enzyme, *Ts*UBE2L3

Purified SP were separated by SDS-PAGE and visualised using colloidal-coomassie staining (Fig 1B) prior to manual excision and analysis by LC/MS/MS. Data was then searched against the *T*. *spiralis* UniProt proteome, its reverse complement, common contaminants of SDS-PAGE and mass spectrometry as well as the rat UniProt proteome. Less than 1% of the protein matches were rat proteins. To validate the data, the experiment was repeated. Peptides from both the first and second experiment were combined and assembled into proteins. The data was cross-referenced with a previous study of the *T*. *spiralis* secretome by Robinson *et al*. identifying all the same proteins, plus many new ones[17–19]. Proteins previously identified in *T*. *spiralis* SP using alternative methods were also present amongst the results[20–25]). To avoid contamination with proteins released from dead or dying parasites, uncoiled and floating *T*. *spiralis* larvae were removed prior to culturing. In addition, to ensure a low level of parasite death, larvae were cultured for 24 h only, before supernatant was collected for SP purification.

One Ub enzyme match was made to a putative protein: ‘ubiquitin-conjugating enzyme E2 L3 (fragment)’. This sequence is annotated as an incomplete open reading frame (ORF) for a UBE2L3 ortholog (UniProt E5S8T6/GI:339240047/ T.sp_00154). UBE2L3, also known as UbcH7, E2-F1, UbcM4, L-UBC and UBCE7, is a UBCc domain Ub conjugating enzyme [26,27]. The annotated fragment ORF for *Ts*UBE2L3 in the database is 145 amino acids in length and lacks a start codon. The UBCc domain of the putative *Ts*UBE2L3 spans the fragment from the first amino acid to the 140th amino acid. Peptides matching *Ts*UBE2L3 were found in the segment of the gel annotated in Fig 1B, corresponding to its predicted size of 17kDa. *Ts*UBE2L3 was predicted to have no signal peptide (signalP, iPSORT), and to be located primarily in the cytoplasm with trace amounts in peroxisomes, nucleus and extracellular space (WolfPSORT scores of 23, 3, 2 and 2, respectively)[28–30]. All protein matches labeled as ‘uncharacterized’ were further analyzed using BLAST and SMART, and no other Ub enzyme domains were identified.

### *Ts*UBE2L3 is responsible for the Ub conjugation activity in *T*. *spiralis* SP

**Fig 2:**
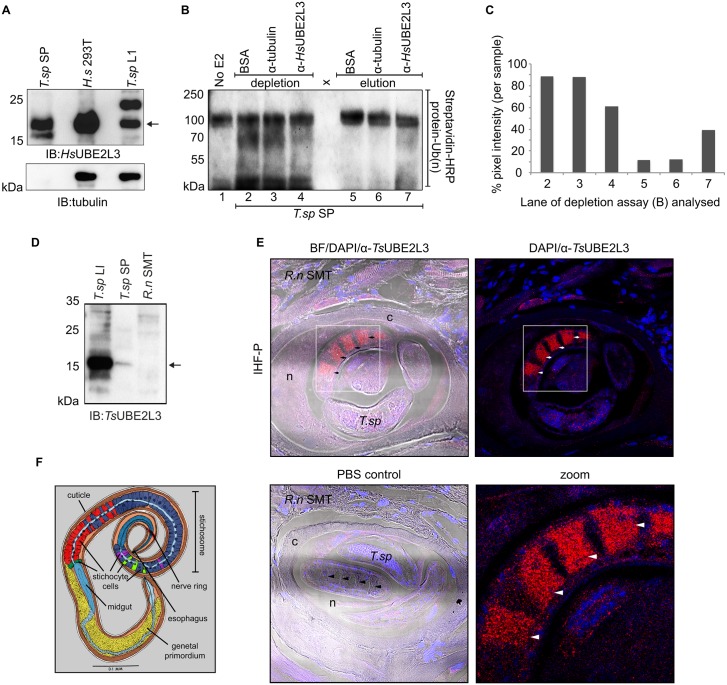
Verification of expression, secretion, activity and localization of TsUBE2L3. **A**. *T*.*sp* SP, HEK 293T cell lysate and *T*. *spiralis* muscle larvae lysate were separated by SDS-PAGE and immuno-blotted with anti-*Hs*UBE2L3 and anti-tubulin antibodies. **B.** Auto-ubiquitination of parkin with Ub-biotin was probed by streptavidin-HRP blot. Reactions from left to right: lane 1: human E1 (UBE1A), E3 (parkin) and Ub-biotin only (no E2). Reactions in lanes 2–4 included human E1, parkin and Ub-biotin, with the following E2 substitution: lane 2: *T*.*sp* SP after resin-bound BSA depletion (BSA), lane 3: *T*.*sp* SP after resin-bound anti-tubulin depletion (α-tubulin), lane 4: *T*.*sp* SP after resin-bound α-*Hs*UBE2L3 depletion (α-*Hs*UBE2L3). In lanes 5–7 the elution fractions from the depletions shown in lanes 2–4 were used to substitute E2. **C**. The pixel intensity of lanes 2–7 of the depletion assay (B) was analysed using an ImageJ gel analysis plugin. For each of the three samples tested in the assay (BSA, α-tubulin and α-*Hs*UBE2L3) the sum of the pixel intensity of the depletion plus elution lanes was taken as 100% (intensity of lane 2 + 5 for BSA and so on). The relative percentages of the depletion and elution lanes were then calculated (intensity of lane 2/(2+5) x 100) and plotted. **D**. A *Ts*UBE2L3-specific antibody was made and its specificity was assessed by immuno-blot against *T*.*sp* L1 lysate, *T*.*sp* SP and rat (*R*.*n*) skeletal muscle tissue (SMT) lysate. The arrow indicates the expected size of *Ts*UBE2L3. **E.** *T*. *spiralis* infected rat skeletal muscle tissue (R.n SMT) was sectioned and analyzed by immuno-histofluorescence. A single *T*.*sp* L1 inside the nurse cell (n), surrounded by a collagen capsule (c) is displayed. Tissue was probed with anti-*Ts*UBE2L3 antibodies (Alexa-568, red) and nuclei were stained using DAPI (blue). Brightfield (BF)/DAPI/anti-*Ts*UBE2L3 and DAPI/anti-*Ts*UBE2L3 merged images are displayed. As a control, infected *R*.*n*SMT was probed with PBS and DAPI only. Arrows indicate stacks of stichocyte cells in the stichosome. **F.** Annotated diagram of *T*. *spiralis* L1 morphology. Image adapted and reprinted from Trichinosis in Man and Animals by S.E. Gould under a CC BY license, with permission from Charles C. Thomas Publisher LTD, original copyright 1970.

The putative *T*. *spiralis* UBE2L3 sequence contains 62.3% of the same residues as the human UBE2L3. A commercially available antibody raised against a region of the human enzyme with high identity to the *T*. *spiralis* protein was used to analyze *T*. *spiralis* SP by immuno-blot for the presence of UBE2L3. Lysate of *T*. *spiralis* muscle larvae and human HEK 293T cells were also analyzed as positive controls (Fig 2A). In HEK 293T lysate one prominent band was observed, corresponding to the predicted size of the human UBE2L3 isoform 1, which is 17.9 kDa in size[31]. In the lysate of *T*. *spiralis* muscle larvae, two prominent bands between 15 and 25 kDa were observed. In the *T*. *spiralis* SP, the most prominent band was observed between 15 and 25 kDa, matching the smaller band in the *T*. *spiralis* lysate, and the only band in HEK 293T lysate. To test for contaminant proteins released from dead or dying larvae, the samples were reacted with anti-tubulin antibodies. Although present in the *T*. *spiralis* and human cell lysates, tubulin was not detected in *T*. *spiralis* SP by immuno-blot or by proteomic analyses of *T*. *spiralis* SP.

In order to determine the contribution of *Ts*UBE2L3 to the Ub conjugation activity of *T*. *spiralis* SP, an *in vitro* depletion assay was carried out (Fig 2B). Using resin-bound anti-UBE2L3 antibodies, SP were depleted of *Ts*UBE2L3 using the immuno-precipitation compatible anti-*Hs*UBE2L3 antibodies. As controls, *T*. *spiralis* SP were depleted using resin-bound BSA and resin-bound anti-tubulin antibodies. Depleted SP samples were then reacted with human parkin in an *in vitro* Ub conjugation assay whereby parkin auto-ubiquitination was analyzed by streptavidin blot. Reduced signal representing Ub conjugation activity was generated by the *Ts*UBE2L3-depleted SP compared to the BSA and anti-tubulin-depleted SP, although signal was not fully depleted due to affinity inefficiency of the antibody. When quantified this amounted to 61% of the total signal from the anti-*Hs*UBE2L3 sample indicating a 3^rd^ of the E2 activity having been depleted (Fig 2C). Signal was also present in the SP proteins that were eluted from the anti-*Hs*UBE2L3 resin, amounting to the remaining 31% of the total sample signal. Very faint signal was observed in the proteins eluted from the BSA or tubulin resin that (at 11 and 12% of the total, respectively) could indicate background or non-specific binding to the resin. Results show that *Ts*UBE2L3 is responsible for at least a 3^rd^ of the Ub conjugation activity observed in *T*. *spiralis* SP (Fig 1A).

### *Ts*UBE2L3 specifically localizes to the *T*. *spiralis* secretory organ during intracellular-stage infection

In order to analyze *Ts*UBE2L3 expression within infected rat skeletal muscle cells (nurse cells) *in situ*, an antibody was developed that could not cross-react with mammalian UBE2L3. Using structural modeling of *Ts*UBE2L3, an N-terminal peptide showing low homology to the corresponding region of the mammalian ortholog (QWRGLLLPDKEPYC) was synthesized and used to raise a *Ts*UBE2L3-specific antibody (Fig 2D). To confirm *T*. *spiralis*-specific reactivity, and absence of mammalian cross-reactivity, anti-*Ts*UBE2L3 was reacted with *T*. *spiralis* lysate, *T*. *spiralis* SP and rat (*rattus norvegicus*-*R*.*n*) skeletal muscle tissue (SMT) lysate. Signal at the expected size between 15 and 25 kDa was observed in the *T*. *spiralis* samples, but not in the *R*.*n*SMT lysate. Histological sections of infected rat skeletal muscle tissue were prepared for immuno-histofluorescence (IHF) analyses using anti-*Ts*UBE2L3 (Fig 2E). Sections were reacted with either anti-*Ts*UBE2L3 or PBS and analyzed by confocal microscopy in order to locate *Ts*UBE2L3 within the nurse cell complex. *Ts*UBE2L3 was clearly and specifically localized to condensed, discrete stacks of secretory cells (stichocytes) inside the secretory organ (stichosome) of each *T*. *spiralis* larva within nurse cells (Fig 2E and 2F).

### *Ts*UBE2L3 stably expresses in mouse skeletal myotubes and specifically interacts with the mammalian E3 ARIH2

**Fig 3:**
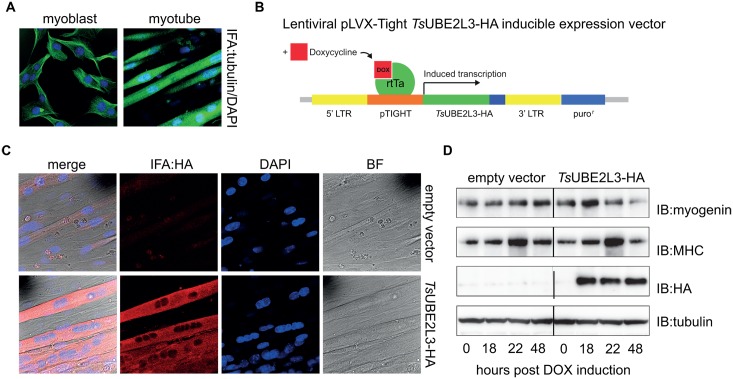
Development of expression system for TsUBE2L3 analysis in C2C12 myotubes. **A**. Wild-type C2C12 undifferentiated myoblasts and terminally differentiated myotubes were probed by immuno-fluorescence (IFA) with anti-tubulin antibodies (Alexa-488, green) and nuclei were stained with DAPI (blue). **B.** Schematic of the pLVX expression construct containing the coding sequence for *Ts*UBE2L3-HA, showing the mechanism of induction by doxycycline (DOX). **C**. Myotubes transduced with empty vector pLVX or *Ts*UBE2L3-HA pLVX and induced with DOX for 24 h were probed by IFA with anti-HA antibodies (Alexa-568, red); nuclei were stained with DAPI (blue). **D**. Empty vector and *Ts*UBE2L3-HA cell lysates were reacted with myogenic differentiation markers, myogenin and myosin heavy chain II (MHC), and anti-HA antibodies, at indicated time points after 24 h DOX induction and analyzed by immuno-blot. The same samples were probed with anti-tubulin as a loading control.

In order to identify potential *Ts*UBE2L3-protein interactions in skeletal muscle cells, transgenic mouse muscle cell (C2C12) lines were generated[32,33]. Since *T*. *spiralis* infects fully differentiated skeletal muscle cells, it was important to investigate the effects of *Ts*UBE2L3 on myotubes (rather than myoblasts) in culture. The full ORF of *Ts*UBE2L3 was confirmed by RACE-PCR analysis (S1, S2 and S3 Figs) and cloned with a hemagglutinin epitope tag into a lentiviral vector, facilitating doxycycline (DOX)-controlled, inducible transgene expression. C2C12 myoblasts were lentivirally transduced with *Ts*UBE2L3-HA, followed by drug selection to create a silent but stable myoblast cell line. In parallel, control transgenic myoblast lines were developed: 1) an empty vector, 2) eGFP-HA, for overexpression of an unrelated protein, and 3) *Mm*UBE2L3-HA, for overexpression of the mouse UBE2L3 ortholog under the same promoter. All cell lines were differentiated into myotubes (Fig 3A) before transgene expression was induced (Fig 3B). *Ts*UBE2L3-HA expression was confirmed by anti-HA immuno-fluorescence (IFA) (Fig 3C) and immuno-blot (IB) after 24 hours (h) of transgene expression (Fig 3D). Reactivity representing *Ts*UBE2L3-HA was observed at the expected size of approximately 17 kDa at 18, 22 and 48 h post-DOX induction, but not before DOX induction (0 h). No reactivity was observed in the empty vector control. IFA analysis also confirmed that in myotubes, *Ts*UBE2L3-HA is cytoplasmic but not nuclear, despite being observed in both the cytoplasm and nucleus of undifferentiated myoblasts (S4 Fig). Myotubes expressing *Ts*UBE2L3-HA did not appear morphologically distinct from the empty vector myotubes (S5 Fig).

**Fig 4:**
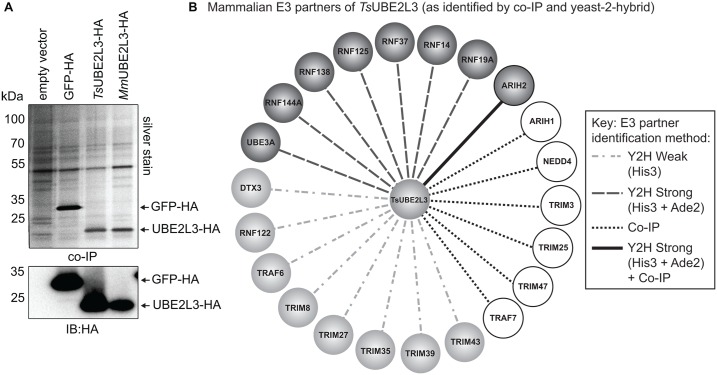
Co-immuno-precipitation (co-IP) and yeast-2-hybrid (Y2H) analyses of TsUBE2L3. **A.** Transgenic C2C12 myotube lines (empty vector, eGFP-HA, *Ts*UBE2L3-HA and mouse *Mm*UBE2L3-HA) after 24 h DOX induction were probed by co-IP with anti-HA antibody. Co-IP elutions were silver-stained and all co-IP'd proteins were analyzed by LC/MS/MS. The same samples were reacted with anti-HA antibodies by immuno-blot (IB) as a control for transgene expression. **B**. Protein interaction network showing positive interactions between *Ts*UBE2L3 and human E3 ligases as observed by yeast-2-hybrid (Y2H) and co-IP analyses. Key indicates if interactions were found by Y2H to be strong, weak or by co-IP.

Using anti-HA antibodies, co-immuno-precipitation (IP) from myotubes of all four transgenic cell lines (empty vector, eGFP-HA, *Ts*UBE2L3-HA and *Mm*UBE2L3-HA) was carried out after 24 h of transgene expression. Proteins that co-precipitated with the HA-tagged bait protein were separated by SDS-PAGE, visualized by silver staining, anti-HA IB (Fig 4A) and analyzed by LC/MS/MS. Data was then searched against the mouse (*Mus musculus*) UniProt proteome, its reverse complement and common contaminants. To validate the data, the experiment was repeated and the two datasets were merged. All proteins co-precipitated from each cell line were cross-referenced. Amongst these, six RING E3 enzymes were identified in the *Ts*UBE2L3 sample, ARIH1, ARIH2, TRIM3, TRIM25, TRIM47 and TRAF7 and one HECT E3, NEDD4 (Fig 4B, Table 1 and S2 Table). As validation for successful *Ts*UBE2L3-specific co-IP, expected “reference” proteins were also identified including the bait protein sequence itself (*Ts*UBE2L3), and the mouse UBE1A (S2 Table). All E3 proteins that co-precipitated with *Ts*UBE2L3-HA (as well as controls) and all proteins that only co-precipitated with *Ts*UBE2L3-HA (and neither control) can be found in S2 Table.

**Table 1:** Identification of TsUBE2L3 E3 interaction partners by co-IP and yeast-2-hybrid (Y2H) analyses. Only interactions measured by Y2H as being strong are listed. Known ubiquitination substrates found by UbiScan analysis to be significantly and specifically upregulated (upreg.) or downregulated (downreg.) after expression of *Ts*UBE2L3-HA are listed.

#	Mammalian E3 ligase			Identification method		E3 domain	Known Ub substrates	Human UBE2L3 interaction (literature)
	Protein name	Human protein ID (Uniprot)	Gene name(s)	Y2H strong	Co-IP			
1	ARIH1	Q9Y4X5	ARIH1, ARI, MOP6, UBCH7B, HUSSY-27, **Ariadne-1**		+	RBR	E1F4E2	+
**2**	**ARIH2**	**O95376**	**ARIH2, ARI2, TRIAD1, HT005, Ariadne-2**	**+**	**+**	**RBR**	**IκBβ**	**+**
3	NEDD4	P46934	NEDD4, NEDD4-1		+	HECT	IGF1R, FGFR1, TNK2, ebola virus VP40	+
4	RNF125	Q96EQ8	RNF125	+		RING	p53, p73	+
5	RNF138	Q8WVD3	RNF138, NARF, HSD-4, HSD4	+		RING	TCF/LEF	+
6	RNF14	Q9UBS8	RNF14, ARA54, HRIHFB2038	+		RBR	unknown	+
7	RNF144A	P50876	RNF144A, KIAA0161, RNF144, UBCE7IP4	+		RBR	PRKDC	+
8	RNF19A			+		RBR	SNCAIP, CASR, SOD1	+
9	RNF37	O94941	UBOX5, KIAA0860, RNF37, UBCE7IP, UIP5	+		RING	unknown	+
10	TRAF7	Q6Q0C0	TRAF7, RNF119, TNF-Rc associated-7, RFWD1		+	RING	unknown	+
11	TRIM3	O75382	TRIM3, BERP, RNF22, RNF97		+	RING	GKAP/ SAPAP1	
12	TRIM25	Q14258	TRIM25, EFP, RNF147, ZNF147		+	RING	DDX58	
13	TRIM47	Q96LD4	TRIM47, RNF100, GOA		+	RING	unknown	

To further investigate the potential for *Ts*UBE2L3 to interact with human E3-RING proteins, a targeted yeast-2-hybrid (Y2H) interaction screen was performed against a collection of 166 human E3-RING proteins (including one HECT domain ligase E6AP/UBE3A) as previously described (S6 Fig and S1 Table)[34,35]. This analysis identified a range of potential *Ts*UBE2L3 interaction partners, observed under low or high stringency conditions (Fig 4B and Table 1). Interactions detected by His3 selection only represent weaker or more transient interactions, while partners detected under combined His3/Ade2 selection represent potentially stronger binary interactions. Notably, a significant proportion of strong, binary interactions were made with members of the non-canonical RING-between-RING (RBR) ligase family (5 out of 13). As the interaction between *Ts*UBE2L3 and the human ARIH2 E3-RBR ligase (also known as ARI1, TRIAD1, Ariadne homologue 2 and All-Trans Retinoic Acid Inducible RING Finger) was detected in both Y2H and co-IP studies, and was the most abundant E3 to co-precipitate specifically with *Ts*UBE2L3 (S2 Table), this high confidence binary complex was therefore selected for further functional characterization.

### *Ts*UBE2L3 interacts with RBR ligases with higher affinity than mammalian orthologs

**Fig 5:**
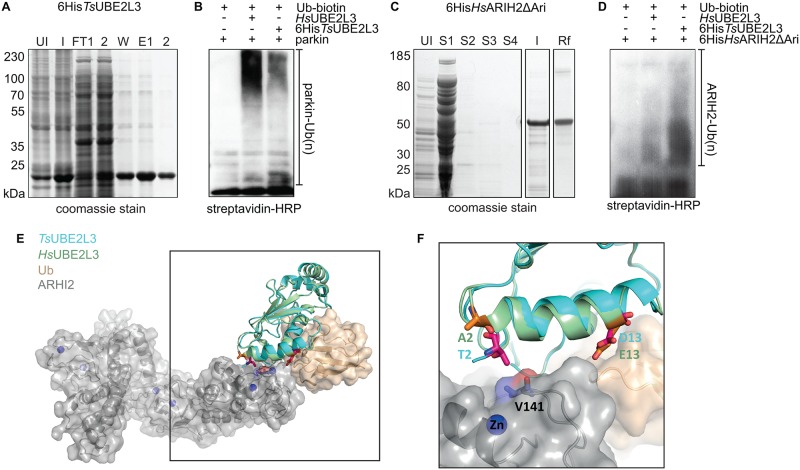
Expression, purification and activity of 6His-TsUBE2L3 and 6His-ARIH2 *in vitro*. **A**. Coommassie stain of 6His-*Ts*UBE2L3 from un-induced (UI) and induced (I) *E*.*coli* cultures, nickel purification resin flow-through (FT1-2), wash (W) and elutions (E1-2). **B and D**. Streptavidin-HRP blots of *in vitro* parkin auto-ubiquitination reactions using human E1 (UBE1A), human Ub-biotin and either no E2, human E2 (*Hs*UBE2L3) or 6His-*Ts*UBE2L3 with **(B)** human parkin as the E3 or **(C)** human ARIH2 as the E3. **C.** Coomassie stain of 6His-*Hs*ARIH2ΔAri from un-induced (UI) *E*.*coli* cultures, inclusion body supernatants (S1-4) induced inclusion bodies (I) and refolded from inclusion bodies (Rf). **E.** Surface representation of ARIH2 (grey) and Ub (wheat) with *Hs*ARIH2 (green) and *Ts*UBE2L3 (cyan) bound to the RING1 domain of ARIH2. Residue differences between the E2-E3 interfaces are shown as sticks (*Hs*ARIH2 in orange and *Ts*UBE2L3 in magenta). Zinc ions are shown as blue spheres, and Val141 of ARIH2 is shown as sticks. **F**. Zoom of the ARIH2:E2:Ub interface.

Recombinant 6His-*Hs*ARIH2ΔAri (lacking the auto-inhibitory Ariadne domain) and 6His-*Ts*UBE2L3 (Fig 5A and 5C) were individually expressed, purified and used for *in vitro* auto*-*ubiquitination assays. Recombinant 6His-*Ts*UBE2L3 Ub conjugation activity was validated using human parkin (Fig 5B). Interestingly, when reacted at the same concentrations, *Ts*UBE2L3 showed a preference (over human UBE2L3) to catalyze lower forms of parkin-Ub (i.e. mono- and di-Ub) and to lead to more overall Ub conjugation of ARIH2 of which the majority was also observed to be lower form ubiquitination (Fig 5B and 5D). This ubiquitination pattern coupled with the selective co-precipitation of ARIH2 with *Ts*UBE2L3 led us to speculate that the worm E2 may bind ARIH2 (and possibly other RBR ligases) with a higher affinity than the mammalian ortholog. To test this hypothesis, we generated structural models to investigate the binding affinity of ARIH2 to both *Ts*UBE2L3 and *Hs*UBE2L3.

Modeling the interactions between *Ts*UBE2L3 and *Hs*UBE2L3 with HsARIH2 and Ub revealed differences in residues across the respective interfaces. While *Ts*UBE2L3 shares 67% sequence identity to the human *Hs*UBE2L3, within 5 Å of the >450 Å2 interface with *Hs*ARIH2, all residues except for two are conserved (83% identity) (Fig 5E). However, overall small global changes between the *Ts*UBE2L3 and *Hs*UBE2L3 sequences and structures (r.m.s.d of 0.5 Å) lead to the formation of additional inter-molecular hydrophobic interactions. In addition, Thr2 of *Ts*UBE2L3 is able to make significant interactions with Val144 of *Hs*ARIH2, not made by Ala2 of *Hs*UBE2L3. This is interesting, as mutation of the corresponding residue in *Hs*ARIH1 (Ile188) has been shown to abolish interaction with UBE2L3[36]. These observations were consistent with predictions by PISA[37] and mCSM-PPI[38] that *Hs*ARIH2 would bind to *Ts*UBE2L3 with higher affinity than *Hs*UBE2L3, with a difference in predicted Gibb's Free Energy of binding of over 1 kCal/mol. By contrast, the larger interface between *Ts*UBE2L3 and *Hs*UBE2L3 with Ub (>900 Å) is less well conserved, with only 58% identity of residues with 5 Å of the interface. Despite this, overall numbers and types of interactions were consistent between the models, with PISA and mCSM-PPI predicting that they would bind Ub with similar binding affinities (difference in predicted Gibb's free energy of binding 0.1 kCal/mol).

### Expression of *Ts*UBE2L3 in skeletal muscle cells leads to a downregulation of ubiquitination, markedly of motor, sarcomere and ECM proteins

**Fig 6:**
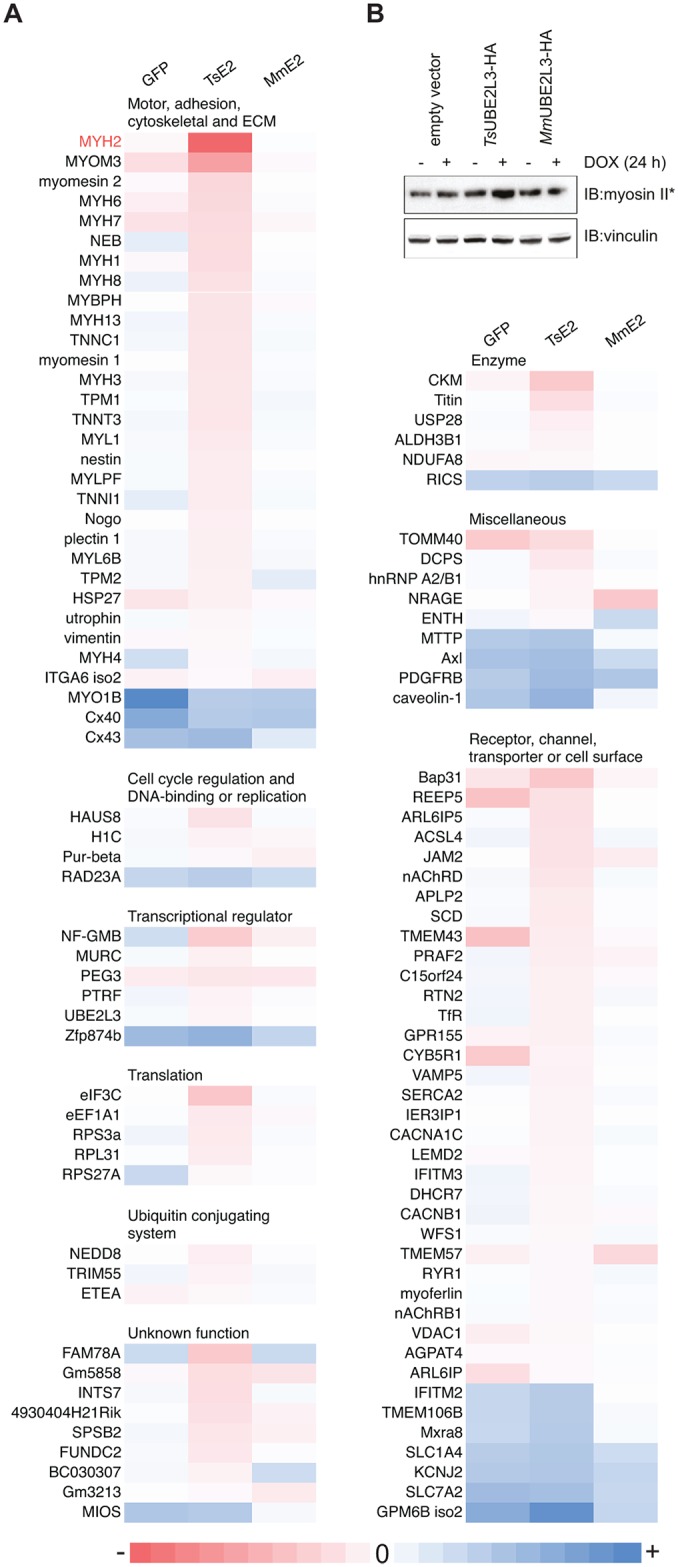
UbiScan analysis of TsUBE2L3 effect on the myotube ubiquitome. **A**. Heatmap displaying proteins in which ubiquitinated peptides were found to be upregulated (blue) or downregulated (red) as measured by a fold change in relation to the empty vector C2C12 myotube cell line after 24 h expression of eGFP-HA (GFP), *Ts*UBE2L3-HA (TsE2) or *Mm*UBE2L3-HA (MmE2). Proteins listed showed a maximum % CV of 49.9 or less, a maximum intensity of 200,000 or more and a fold change in ubiquitination of 2.5 or more in response to *Ts*UBE2L3-HA expression only. Where multiple ubiquitinated peptides were identified for the same protein (according to the protein description as assigned by Cell Signaling Technology), the mean fold change was calculated. Proteins were grouped according to annotated biological ontology. **B**. Myosin II/fast skeletal myosin (MYH2) (the protein with the largest significant fold change specifically in response to *Ts*UBE2L3 expression) was analyzed by immuno-blot in the empty vector and *Ts*UBE2L3 C2C12 myotube cell lines before and after 24 h of induction (DOX) using anti-myosin II antibodies and anti-vinculin as a loading control.

In order to assess the global effect of *Ts*UBE2L3 on C2C12 myotubes, a ubiquitome analysis was carried out using a post-translational modification (PTM) UbiScan (Ub remnant proteomics) method[39,40]. This method employs the K-ε-GG antibody that binds to the di-Gly motif that remains on a ubiquitinated residue of a trypsin-digested protein (a Ub remnant peptide). This allows enrichment of ubiquitinated peptides from a whole cell lysate for LC/MS/MS analysis. Transgene expression was induced in all four mouse myotube cell lines (empty vector, eGFP, *Ts*UBE2L3 and *Mm*UBE2L3) for 24 h before myotubes were harvested for analysis. Searches were performed against the mouse UniProt proteome and peptide matches were quantified and normalized. To calculate fold change, the abundance and strength of each protein match in a particular sample (eGFP, *Ts*UBE2L3 and *Mm*UBE2L3) was compared to the control sample (empty vector). Results for all three cell lines were filtered to contain only those with a significant fold change of 2.5 or above in response to *Ts*UBE2L3, with a maximum intensity of 200,000 or above and a maximum % CV of 49.9 or below. Results were categorized based on annotated biological ontology for further analysis (Fig 6A and S3 Table). The data was screened for changes in ubiquitination of any of the known mammalian substrates of all strong Y2H E3 partners or E3s that were identified by co-IP (whether known interactors of UBE2L3 or not). None were identified, suggesting an alternate role of TsUBE2L3 to that of its host ortholog.

The ontological group that, overall, displayed the largest specific fold-change in response to *Ts*UBE2L3 expression was composed of proteins known to play a role in motility/contraction, sarcomere structure, extracellular matrix (ECM) and cytoskeleton such as myosin, myomesin, nebulin and troponin. Notably, the majority of proteins that displayed a significant change in ubiquitination in response the *Ts*UBE2L3 expression showed a negative fold-change, i.e. in the *Ts*UBE2L3 column of the heatmap (Fig 6A) fewer ubiquitinated forms of the proteins are observed. This suggests that in the presence of *Ts*UBE2L3, they were either deubiquitinated or not ubiquitinated to begin with (stabilized). We therefore hypothesized that the consequence of this would be observed as a reduction in ubiquitination and a resulting increase in abundance. No significant difference could be observed in the total amount of ubiquitinated protein in *Ts*UBE2L3 cells compared to empty vector myotubes as measured by pull-down of polyubiquitinated proteins using tandem ubiquitin binding motif entity resin (TUBE2, S7A and S7B Fig). However an increase in the abundance of native myosin II (MYH2) in the *Ts*UBE2L3 myotubes (the protein that showed the largest fold change in ubiquitination specifically in response to *Ts*UBE2L3 expression) was observed by immuno-blot in relation to empty vector cells (Fig 6B and S3 Table). This indicates that there is either a reduction in Ub-mediated proteasomal degradation or an increase in expression of particular proteins that remain unaffected in empty vector or *Mm*UBE2L3 myotubes. Despite an apparent stabilization of proteins important to sarcomere structure, the overall organization of the myotube sarcomere appeared to be disrupted by *Ts*UBE2L3 expression (compared to wild-type, empty vector or *Mm*UBE2L3 myotubes). An IFA assay of the sarcomere Z-disc protein α-actinin, showed a larger proportion of *Ts*UBE2L3 cell structures appearing to be disordered and without characteristic myotube α-actinin-positive stripes. Although quantification is not statistically significant, there appears to be a definite trend of fewer α-actinin-positive stripes in the TsUBE2L3 sample as compared to controls (S7C and S7D Fig).

## Discussion

Various pathogenic viral and bacterial effectors have been identified and characterized as Ub-specific enzymes that successfully manipulate the Ub pathway of the host during infection. Studies in parasites have largely focused on endogenous Ub system components that are important or essential for parasite biology[41–44]). Although these studies demonstrate potential for the identification of novel targets for therapeutic agents, the study of the role of the Ub pathway at the host-parasite interface remains largely uncharted territory. During this study we used *T*. *spiralis* as an effective model to investigate whether there is a role for the Ub pathway in host-parasite interactions. We identified the first parasite-derived, host-targeted E2 enzyme, *Ts*UBE2L3, setting precedence for the further investigation of the role of parasite Ub conjugation enzymes and the Ub pathway in direct host-parasite interactions. E3 activity was also identified in *T*. *spiralis* SP, although the identity of the proteins responsible could not be elucidated from conventional LC/MS/MS using the annotated *T*. *spiralis* proteome. We are currently carrying out further analyses to isolate this activity and identify the protein(s) responsible.

*Ts*UBE2L3 is stored in the secretory organ of *T*. *spiralis* and is released as an active enzyme by muscle-stage (L1) larvae. In isolated *T*. *spiralis* SP, the E1, E3 and/or the substrates required for Ub conjugation by *Ts*UBE2L3 are not present, suggesting that this enzyme requires partner components of the Ub conjugation cascade to be provided by an external source. We therefore hypothesized that only when in contact with host partner proteins (i.e. host E3s) does secreted *Ts*UBE2L3 carry out its intended function as an E2 enzyme. As validation of this hypothesis, a number of potential mammalian E3 ligase partners for *Ts*UBE2L3 were identified. The strongest interaction identified was with the RBR ligase ARIH2. Very little is known about the function of RBR ligases such as ARIH2, and to date only one Ub substrate of ARIH2 has been reported in dendritic cells, the NF-κB inhibitor IκBβ[45]. Although human ARIH2 and UBE2L3 are known to interact, ARIH2 was not identified by co-IP as a partner for the mouse UBE2L3 ortholog in skeletal myotubes. When reacted with human parkin *in vitro*, *Ts*UBE2L3 was observed to catalyze more mono-Ub-parkin than the human UBE2L3. When reacted with human ARIH2 *in vitro*, although both E2s showed a preference for lower Ub forms (over poly-Ub forms) of ARIH2-Ub, *Ts*UBE2L3 used at the same concentration was observed to catalyze overall more ubiquitination than *Hs*UBE2L3.

The current accepted mechanism of RBR-mediated Ub conjugation suggests that a Ub-charged E2 binds to the RING1 domain, Ub is then passed on to the RING2 domain, and then onto the substrate. The E2 must then dissociate to allow a new Ub-charged E2 to bind to catalyze any subsequent Ub conjugation that would lead to either Ub chain formation or substrate exchange[46]. Therefore, we postulate that if an E2 binds an RBR more tightly, formation of mono-Ub or lower forms of Ub would be promoted due to the slower dissociation of E2 from E3, i.e. the substrate would be more likely to dissociate/exchange before the E2. Furthermore, the sequestration of the E3 by the E2, could lead to an overall reduction in activity of the E3. It was therefore hypothesized that in our myotube system the parasite *Ts*UBE2L3 has a higher affinity for ARIH2 than the mouse ortholog[47]. The structural modeling results indeed agreed with this hypothesis, showing that the *T*. *spiralis* E2 makes a more extensive hydrophobic interface with the E3, leading to tighter binding quantified at approximately 1 kCal/mol difference in binding affinities between the human and parasite E2s. It is therefore possible that the parasite UBE2L3 has evolved a higher affinity than the host UBE2L3 in order to outcompete the endogenous enzyme in nurse cells and sequester the host E3 in an inhibitory manner.

On a cellular level, *Ts*UBE2L3 expression in C2C12 myotubes caused a significant downregulation of ubiquitination of motor, sarcomere and ECM-specific proteins; most notably an effect not observed in response to the expression of the mammalian ortholog. Although this presented as an increase (stabilization) in the abundance of native form of myosin II (MYH2—the protein with the largest significant fold change in response to *Ts*UBE2L3 expression), the mechanism and physiological effect of this stabilization is yet to be determined. The remodeling of mammalian skeletal muscle tissue is highly regulated by ubiquitination[48–50]. In addition, nurse cell development during *T*. *spiralis* infection of muscle cells involves dramatic remodeling of the myofibril[51]. Despite previous ultrastructural and biochemical evidence suggesting destruction of myofibrillar proteins in the infected myotube [16,52], the process of nurse cell maintenance is likely very complex. As such, a role for stabilization may be necessary, particularly at later stages of nurse cell development where the L1-stage worm is creating a stable “hideout” within the host cell. Indeed if the regulation of the abundance of the proteins that comprise the sarcomere is essential for proper sarcomere formation, then a significant upregulation in abundance due to a reduction in ubiquitin-mediated turnover may lead to structure disruption as was observed by IFA. Two muscle specific E3 ligases known to be upregulated during muscle remodeling and wasting are Murf1 and atrogin-1. These ubiquitinate structural and motor proteins that are subsequently degraded by the proteasome[53–55]. An interaction between *Ts*UBE2L3 and Murf1 is unlikely given that the mammalian UBE2L3 cannot not interact with Murf1, therefore the loss of myofibrillar proteins seen in previous studies of nurse cells is unlikely to be an effect resulting from *Ts*UBE2L3.

Since *Ts*UBE2L3 is an E2, it was initially surprising to observe less overall ubiquitination in C2C12 cells expressing the parasite protein. However if the mechanism of interaction of *Ts*UBE2L3 with all host E3 partners is the same as that observed with ARIH2 (namely to bind more tightly than and outcompete the host E2), then inhibitory E3 sequestration may play a role in an overall reduction in ubiquitination of target proteins. It is also possible that *Ts*UBE2L3 acts upstream of a different ubiquitination cascade, perhaps targeting another E2 or E3 as a substrate for degradation that would ordinarily ubiquitinate these proteins. It must be noted that studying the effect of *Ts*UBE2L3 on intact myotubes as opposed to already transformed (*T*. *spiralis*-infected) cells may not reflect its true biological role during infection or relevant stage during nurse cell formation. The only way to fully assess this would be to generate *Ts*UBE2L3 knock-out parasites, a process that is currently technically impossible until *T*. *spiralis* is rendered genetically tractable.

Despite a high level of sequence identity, *Ts*UBE2L3 appears to have evolved a different binding behavior and cellular function to its host ortholog. Functional divergence of orthologous proteins is commonly observed to have evolved between non-pathogenic and pathogenic species of the same lineage, and during specialization of the same pathogen to different hosts[56–58]. The evolution of worm genomes to species-specific parasitism has also been reported[59]. It is therefore possible that this E2 of the zoonotic *T*. *spiralis* parasite evolved under pressure to compete with a host ortholog whose identity is highly conserved across a wide range of mammalian species. In summary, we have discovered a novel effect of *Ts*UBE2L3 on skeletal muscle tissue, namely that it suppresses ubiquitination and degradation of skeletal muscle specific proteins, thus having a stabilizing effect. Muscular degenerative diseases often involve the loss of muscle mass, structure and function as a result of the breakdown of proteins such as myosins. The Ub/proteasome system plays a key role in this pathology[60,61]. Since treatment of these disorders could involve the stabilization of these degradative pathways, we are led to speculate on the therapeutic potential of *Ts*UBE2L3. Most parasitic worm-derived therapeutics are products involved in immuno-regulation, since many of these parasites have been found to skew the mammalian immune response towards one that is beneficial in allergic and autoimmune disorders[62–64]. However there are some parasite-derived mammalian cell modulators whose (non-immuno) activity is also being investigated for medical purposes[65]. Given the chronic infections many parasites establish and their specialized tissue tropism within the host, a better understanding of their cell biology could yield insight into novel treatments for unrelated, non-infectious diseases.

## Materials and Methods

For detailed experimental methods and primer sequences please refer to Supplemental Materials and Methods (S1 Text).

### Collection and analysis of *T*.*spiralis* secreted proteins

*T*. *spiralis* L1-stage muscle larvae were isolated from infected rat skeletal muscle tissue (Sprague-Dawley, Harlan UK LTD, Bicester OX25 1TP) by digestion with acidified pepsin and secreted products (SP) were collected as described by Arden et al[66]. Protein concentrations were normalized by BCA assay (Pierce) and either separated by SDS-PAGE and manually extracted from the gel or precipitated by trichloroacetic acid using standard methods.

### LC/MS/MS

Samples were digested with trypsin using standard protocols and peptides were analyzed on either an Orbitrap XL2 (*T*. *spiralis* SP) or Elite (co-IP) mass spectrometer. MS2 spectra were searched using SEQUEST v.28 against a composite database derived from the UniProt *Trichinella spiralis* proteome, its reversed complement and known contaminants. Peptide spectral matches were filtered to either a 1% (*T*. *spiralis* SP) false discovery rate (FDR) or a 1.7% FDR (co-IP) using the target-decoy strategy combined with linear discriminant analysis.

### *Ts*UBE2L3 depletion and Ub conjugation assays

*T*. *spiralis* SP were depleted of *Ts*UBE2L3 by incubating with Dynabead-bound (Life Technologies)-anti-*Hs*UBE2L3 antibodies. Bound proteins were eluted with glycine elution and refolded into Ub assay buffer. For parkin auto-ubiquitination assays, reactions were initiated using the Boston Biochem K105 kit according to the manufacturer’s instructions and p-parkin (kindly donated by Wade Harper and Alban Ordureau, Harvard Medical School)[67]. Reaction mixtures were initiated by addition of biotin-Ub:Ub and incubated at 37°C for 1.5 h. Proteins were separated by SDS-PAGE and analyzed by streptavidin-blot.

### Generation and differentiation of C2C12 cell lines

PLVX Tet On (1 ml) and pLVX Tight Puro lentivirus particles were prepared in HEK 293T cells (ATCC) as described by Mostoslavsky et al[68]. Stable transgenic C2C12 (ATCC) cell lines were generated by spinfection using equal volumes of Tet On and Tight Puro particles added with 8 μg/ml Polybrene (Sigma) to cells at 70% confluency. Stable C2C12 cell lines were drug selected and differentiated into myotubes before transgene induction using 2 μg/ml doxycycline (DOX).

### Preparation of myotube lysates and co-immuno-precipitation

Nuclear and cytosolic extracts were prepared using the CelLyic NuCLEAR Extraction kit (Sigma-Aldrich) according to the manufacturer’s instructions. The cytosolic and nuclear fractions of each sample were pooled and protein concentrations normalized by BCA assay. Proteins were immunoprecipitated using anti-HA affinity matrix (Roche) and HA peptide elution. Supernatants were pooled for SDS-PAGE, silver staining and LC/MS/MS analysis.

### Yeast-2-hybrid (Y2H) analysis

Human E3-RING prey clones were constructed as described previously[34,35] using pACTBD/E-B vectors[69]. The *Ts*UBE2L3 open reading frame was cloned from pGEMTeasy into the bait pGBAE-B Y2H vector through *in vivo* gap repair cloning as previously described[69,70]. The *Ts*UBE2L3 bait clone was mated against arrays of 166 full-length CDS human E3-RING prey clones and 39 prey clones containing the cytoplasmic domains of human transmembrane E3-RING proteins. Growth of positive colonies was monitored and scored over a period of 14 days (S6 Fig and S1 Table).

### UbiScan: LC/MS/MS

UbiScan analysis was carried out by Cell Signaling Technology as previously described[71–73] and LC/MS/MS was carried out on enriched trypsin-digested Ub peptides. MS/MS spectra were evaluated using SEQUEST and the Core platform from Harvard University[39,40,74]. Searches were performed against the most recent update of the NCBI *Mus musculus* database with mass accuracy of +/-5 ppm for precursor ions and 1 Da for product ions. Results were filtered for the presence of the intended motif (K-εGG).

## Supporting Information

S1 Table Yeast-2-hybrid raw plate layout. Layouts of E3-RING prey arrays screened against *Ts*UBE2L3 (see S6 Fig). Top and middle panel contain full-length cds E3-RING prey clones, generated by Markson and Woodsmith et al [34,35]. Bottom panel contains truncated transmembrane E3-RING prey clones (transmembrane domains removed), *Non-E3-RING clones in the array. Red text indicates interaction with *Ts*UBE2L3.
(TIF)

S2 Table Co-IP data. Table of co-IP data showing reference results including the annotated protein sequence that is currently found in the NCBI database (“ubiquitin-conjugating enzyme E2 L3, partial” GI:339240047), all E3 proteins that co-IP'd with *Ts*UBE2L3-HA (as well as controls) and all proteins that only co-IP'd with *Ts*UBE2L3-HA (and neither control). E3 ligases are highlighted in bold font.
(XLSX)

S3 Table Raw UbiScan data. UbiScan data as generated by Cell Signaling Technology showing fold change by protein type in levels of ubiquitinated peptides in GFP, *Ts*UBE2L3 and *Mm*UBE2L3 myotubes, in relation to the empty vector control cell line.
(XLSX)

S1 Text Supplemental Materials and Methods. (DOCX)

S1 Fig Race confirmation of TsUBE2L3 3’ ORF. The RACE-PCR sequencing data of the 3’ end of *Ts*UBE2L3 cDNA aligned with the annotated fragment coding sequence that is currently found in the NCBI database. Figure shows the position of the RACE oligo, the RACE 3’ forward primer-binding site, the stop site of the full coding sequence of the gene, the custom gene-specific forward primer (GSFP)-binding site and the cloning vector (pGEMTeasy) sequences. The sequence contained a 3’ continuation after the stop codon that was identified as *T*. *spiralis* genomic DNA (gDNA).
(TIF)

S2 Fig Race confirmation of TsUBE2L3 5’ ORF. The RACE-PCR sequencing data of the 5’ end of *Ts*UBE2L3 cDNA aligned with the annotated fragment coding sequence that is currently found in the NCBI database. Figure shows the position of the RACE oligo, the RACE 5’ forward primer-binding site, the start site of the full coding sequence of the gene, the custom gene-specific reverse primer (GSRP)-binding site and the cloning vector (pGEMTeasy) sequence.
(TIF)

S3 Fig TsUBE2L3 alignments. The annotated fragment (incomplete) coding sequence (cds) for *Ts*UBE2L3 (GI:339240046/*Ts*p_00154/UniProt: E5S8T6—found in the contig sequence: GI:316975344) that is currently found in the NCBI database was aligned with the full RACE-PCR confirmed sequence from start to stop, compiled from both 5’ RACE-PCR and 3’ RACE-PCR data, and with the human UBE2L3 isoforms #1, #3 and #4 cds’ (GI:4507789, GI:373432682 and GI:373432684 respectively-Uniprot: P68036).
(TIF)

S4 Fig IFA of transgenic TsUBE2L3-HA C2C12 cells. IFA of transgenic *Ts*UBE2L3-HA C2C12 undifferentiated myoblasts and differentiated myotubes showing Alexa-488 conjugated anti-HA (green), DAPI-stained nuclei (blue), brightfield (BF) and overlay (merge of three signals). Row 1 shows myoblasts with cytoplasmic *Ts*UBE2L3-HA localization in a myoblast. Row 2 shows cytoplasmic and nuclear *Ts*UBE2L3-HA localization in myoblasts. Row 3 shows only cytoplasmic *Ts*UBE2L3-HA localization in myotubes. Images are representative of multiple biological replicates.
(TIF)

S5 Fig IFA of transgenic (eGFP-HA and TsUBE2L3-HA) C2C12 cell lines. IFA of anti-tubulin showing no overall morphological/shape change of cells.
(TIF)

S6 Fig Yeast-2-hybrid (Y2H) raw data. Image of Y2H plates (all included in screen-positive and negative). Targeted Y2H matrix mating assays screening *Ts*UBE2L3 against arrays of full length and truncated E3-RING proteins (for layout see S1 Table). Yeast growth indicates positive protein-protein interaction. 0–5 colonies: background yeast growth, 6–20 colonies: weak interaction, 20–200 colonies: medium interaction, full plaque: strong interaction. Interactions observed only with the Ade2 reporter (SD-WLA) are not considered true positive interactions, however those with the His3 reporter only (SD-WLH(AT)) are considered positive.
(TIF)

S7 Fig *A*. *TUBE2 IP from transgenic myotube cell lines*. Anti-Ub immuno-blot showing normalized lysates of empty vector and *Ts*UBE2L3 C2C12 myotube cells (input), results of tandem ubiquitin binging entity pull-down (TUBE2), and unbound protein. An anti-vinculin immuno-blot of the same samples was included as a loading control. *B*. *Quantification of Ub immuno-blot*. ImageJ was used to analyze the intensity (raw pixel area) of each smear from the immuno-blot shown in A. *C*. *IFA of transgenic myotube cell lines*. IFA of anti-α-actinin/Alexa488 (with DAPI-stained nuclei) showing fewer ordered sarcomere A-bands in the *Ts*UBE2L3-HA cells than in the wild-type, empty vector and *Mm*UBE2L3 cells. **D**. ImageJ quantification (using the “analyze stripes” plugin) of the number of striped α-actinin-positive structures in each cell line. The mean number of stripes calculated from the analysis of 7 images (per cell line) taken over 3 independent experiments is displayed for each cell line, with error bars representing the standard error of the mean.
(TIF)
