# Supplementary figures and images for: Ubiquitin-Dependent Modification of Skeletal Muscle by the Parasitic Nematode, Trichinella spiralis
Source: PLoS Pathog. 2016 Nov 21;12(11):e1005977. doi: 10.1371/journal.ppat.1005977 (PMC5117777; doi:10.1371/journal.ppat.1005977)

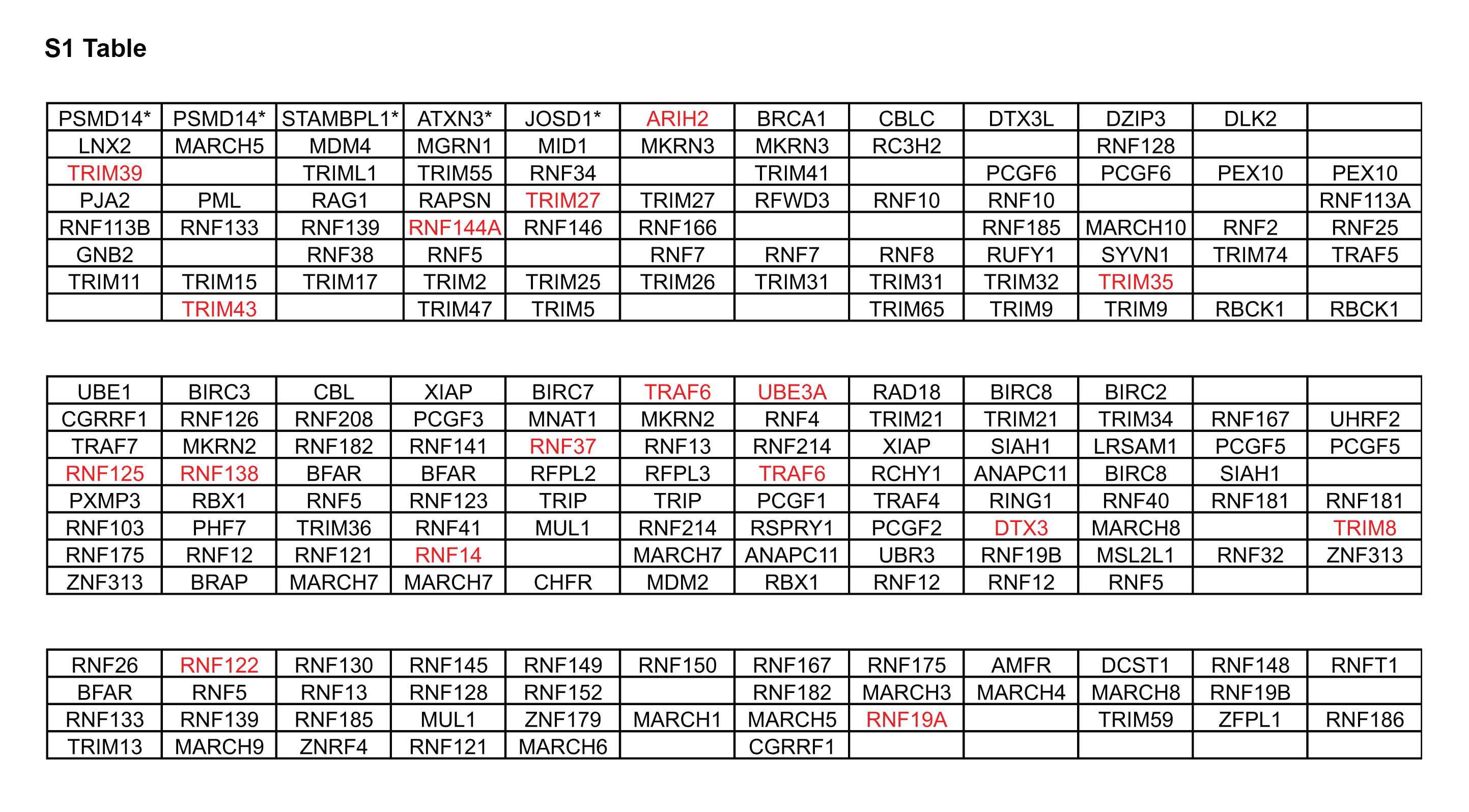

Supplement: S1 Table — Layouts of E3-RING prey arrays screened against TsUBE2L3 (see S6 Fig). Top and middle panel contain full-length cds E3-RING prey clones, generated by Markson and Woodsmith et al [34,35]. Bottom panel contains truncated transmembrane E3-RING prey clones (transmembrane domains removed), *Non-E3-RING clones in the array. Red text indicates interaction with TsUBE2L3. (TIF) [file ppat.1005977.s001.tif]

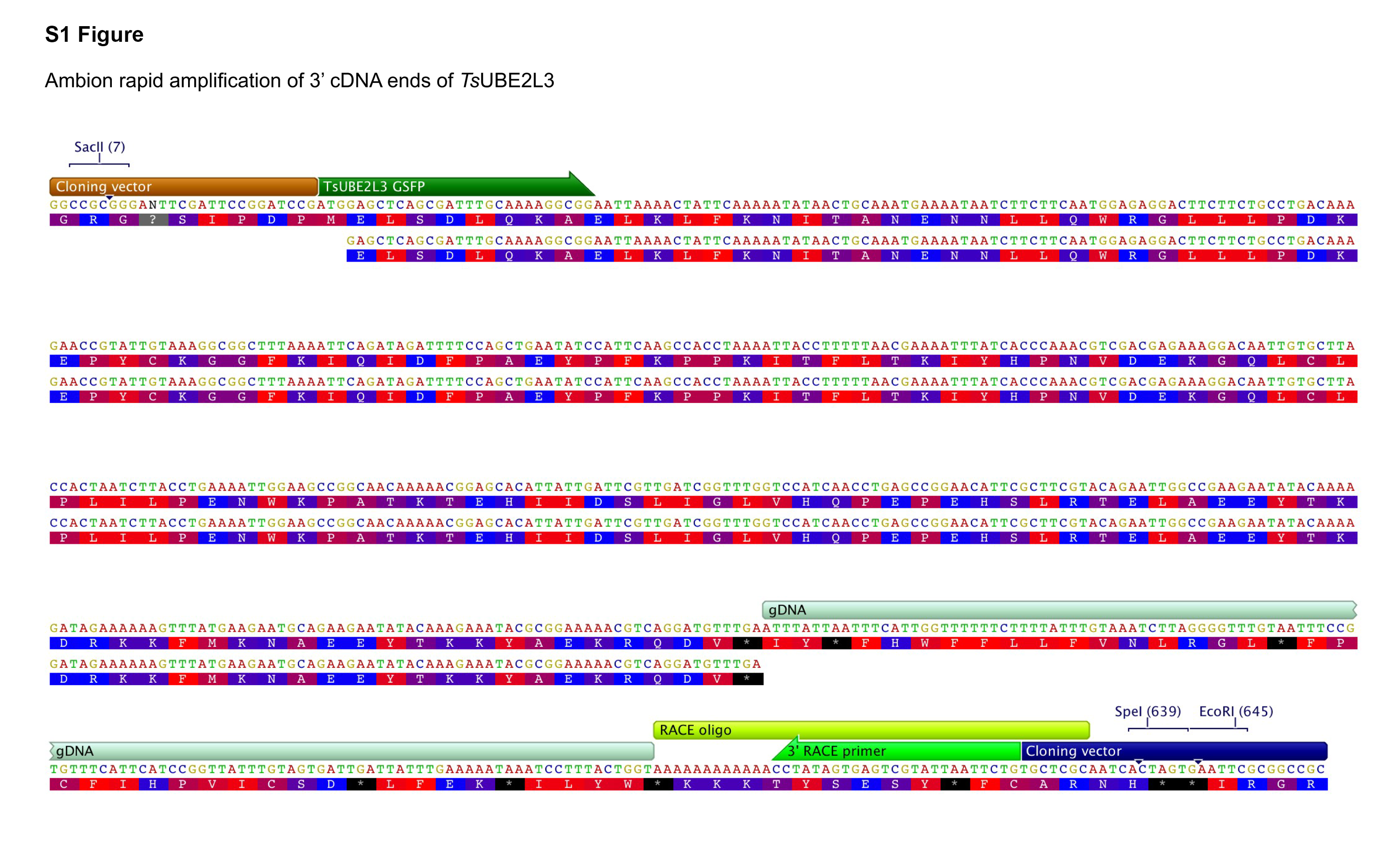

Supplement: S1 Fig — The RACE-PCR sequencing data of the 3’ end of TsUBE2L3 cDNA aligned with the annotated fragment coding sequence that is currently found in the NCBI database. Figure shows the position of the RACE oligo, the RACE 3’ forward primer-binding site, the stop site of the full coding sequence of the gene, the custom gene-specific forward primer (GSFP)-binding site and the cloning vector (pGEMTeasy) sequences. The sequence contained a 3’ continuation after the stop codon that was identified as T. spiralis genomic DNA (gDNA). (TIF) [file ppat.1005977.s005.tif]

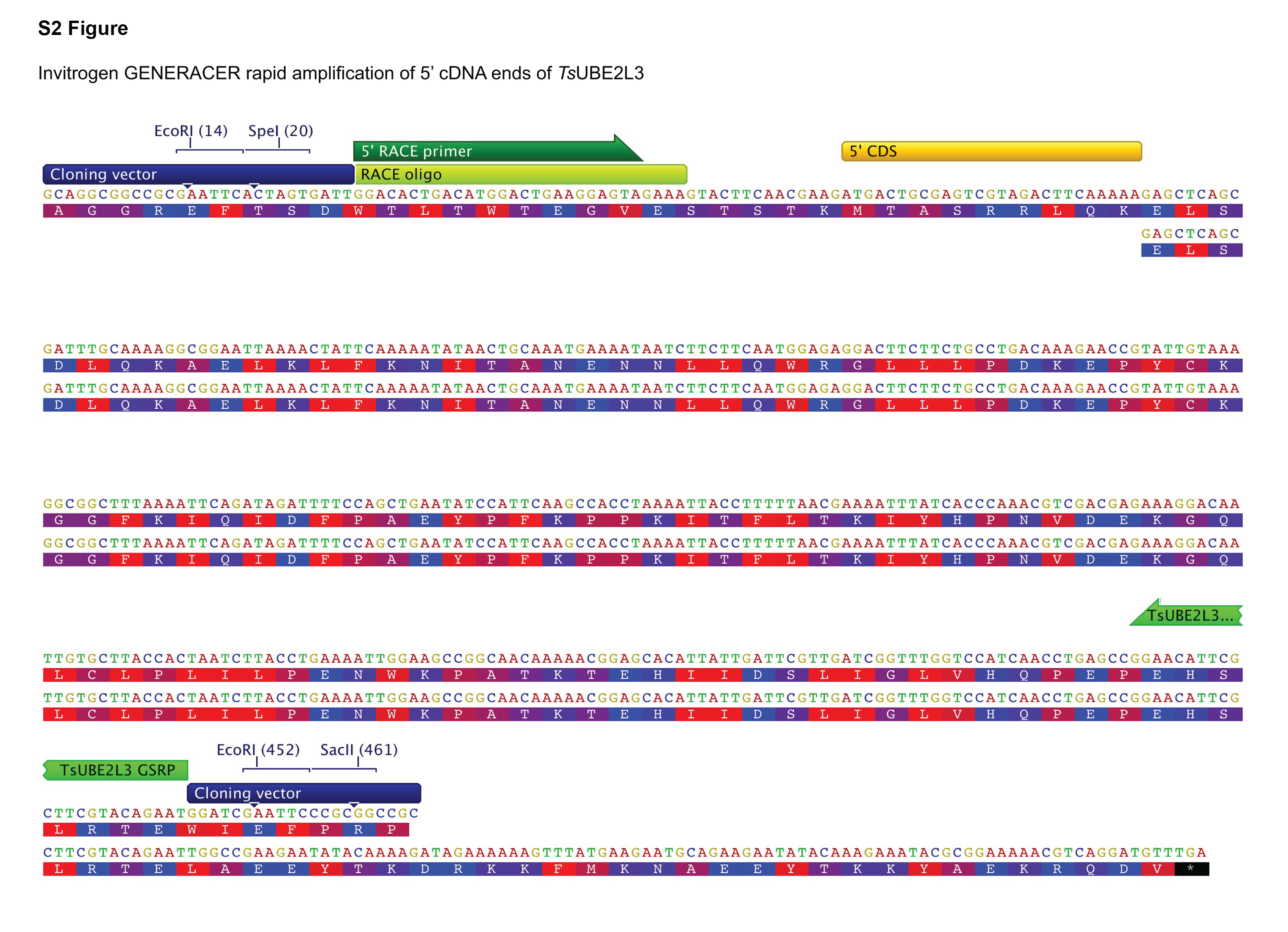

Supplement: S2 Fig — The RACE-PCR sequencing data of the 5’ end of TsUBE2L3 cDNA aligned with the annotated fragment coding sequence that is currently found in the NCBI database. Figure shows the position of the RACE oligo, the RACE 5’ forward primer-binding site, the start site of the full coding sequence of the gene, the custom gene-specific reverse primer (GSRP)-binding site and the cloning vector (pGEMTeasy) sequence. (TIF) [file ppat.1005977.s006.tif]

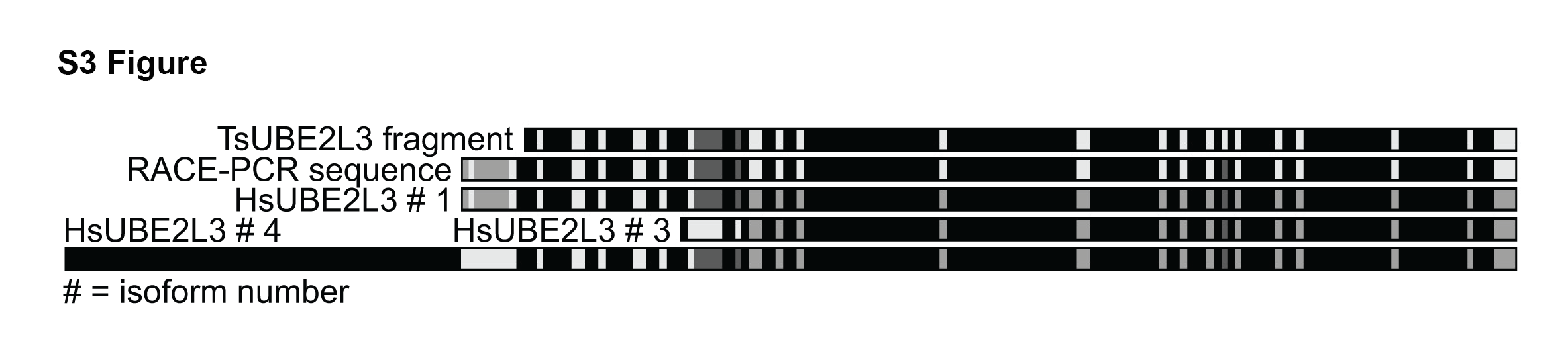

Supplement: S3 Fig — The annotated fragment (incomplete) coding sequence (cds) for TsUBE2L3 (GI:339240046/Tsp_00154/UniProt: E5S8T6—found in the contig sequence: GI:316975344) that is currently found in the NCBI database was aligned with the full RACE-PCR confirmed sequence from start to stop, compiled from both 5’ RACE-PCR and 3’ RACE-PCR data, and with the human UBE2L3 isoforms #1, #3 and #4 cds’ (GI:4507789, GI:373432682 and GI:373432684 respectively-Uniprot: P68036). (TIF) [file ppat.1005977.s007.tif]

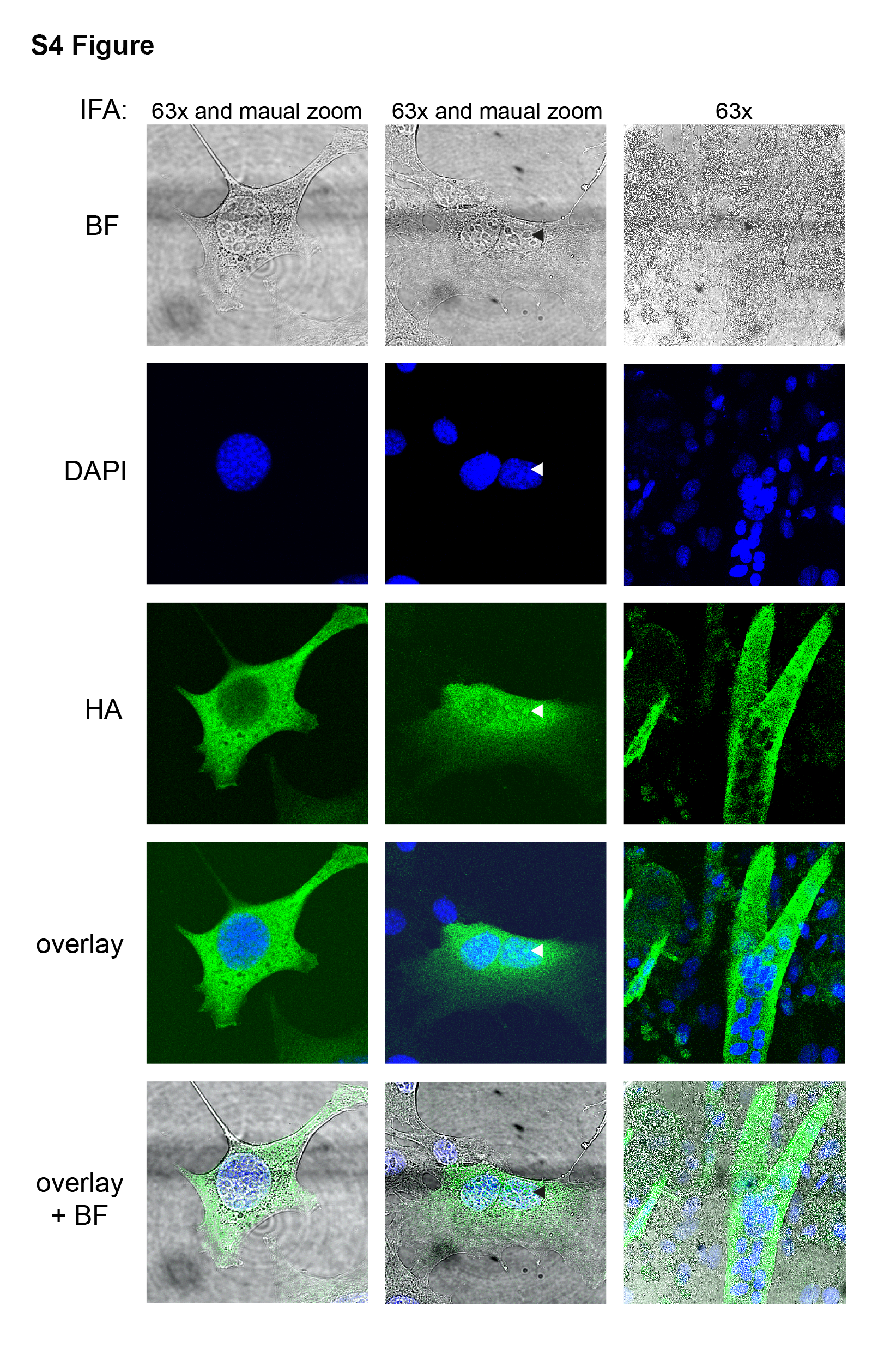

Supplement: S4 Fig — IFA of transgenic TsUBE2L3-HA C2C12 undifferentiated myoblasts and differentiated myotubes showing Alexa-488 conjugated anti-HA (green), DAPI-stained nuclei (blue), brightfield (BF) and overlay (merge of three signals). Row 1 shows myoblasts with cytoplasmic TsUBE2L3-HA localization in a myoblast. Row 2 shows cytoplasmic and nuclear TsUBE2L3-HA localization in myoblasts. Row 3 shows only cytoplasmic TsUBE2L3-HA localization in myotubes. Images are representative of multiple biological replicates. (TIF) [file ppat.1005977.s008.tif]

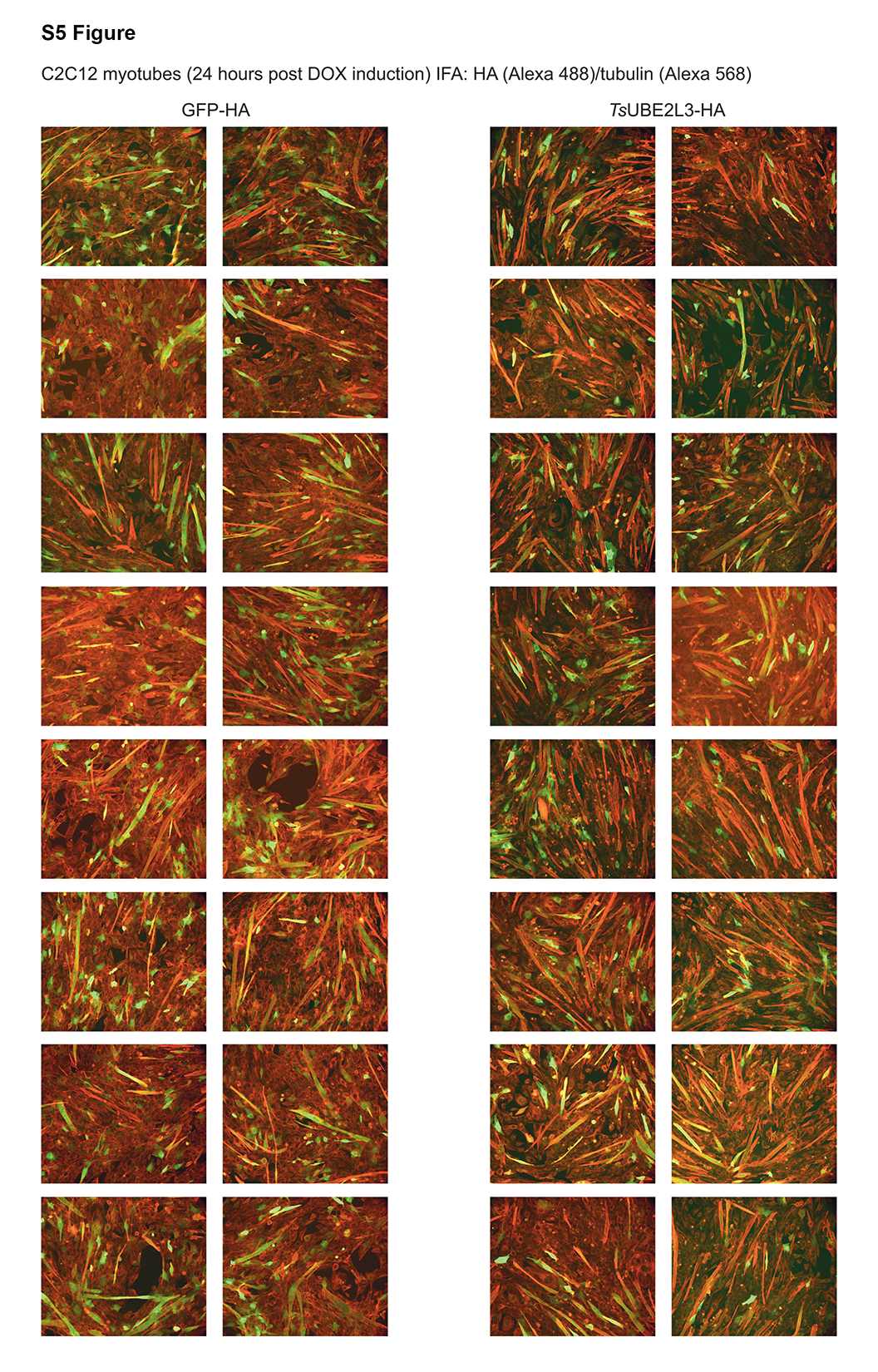

Supplement: S5 Fig — IFA of anti-tubulin showing no overall morphological/shape change of cells. (TIF) [file ppat.1005977.s009.tif]

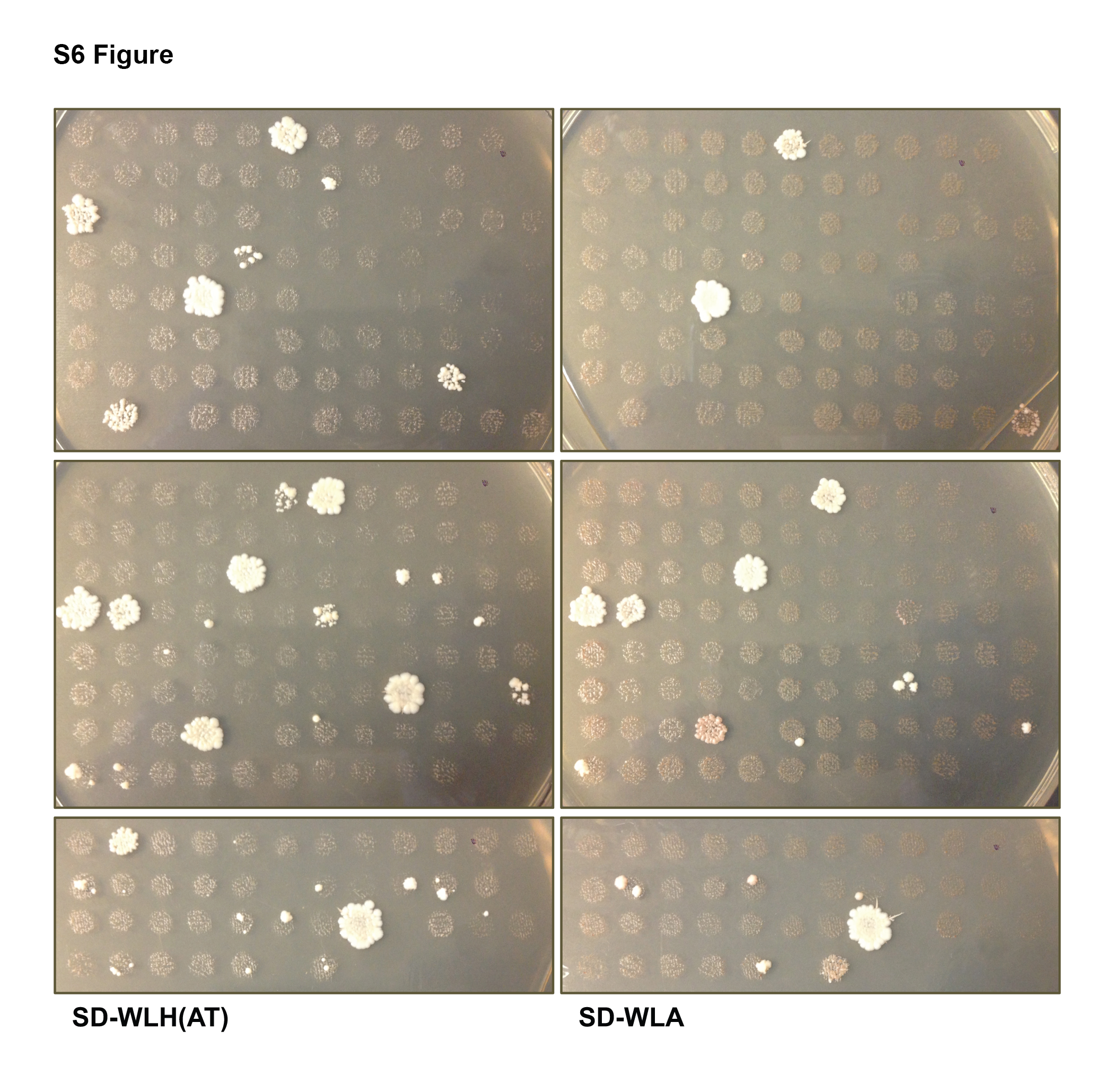

Supplement: S6 Fig — Image of Y2H plates (all included in screen-positive and negative). Targeted Y2H matrix mating assays screening TsUBE2L3 against arrays of full length and truncated E3-RING proteins (for layout see S1 Table). Yeast growth indicates positive protein-protein interaction. 0–5 colonies: background yeast growth, 6–20 colonies: weak interaction, 20–200 colonies: medium interaction, full plaque: strong interaction. Interactions observed only with the Ade2 reporter (SD-WLA) are not considered true positive interactions, however those with the His3 reporter only (SD-WLH(AT)) are considered positive. (TIF) [file ppat.1005977.s010.tif]

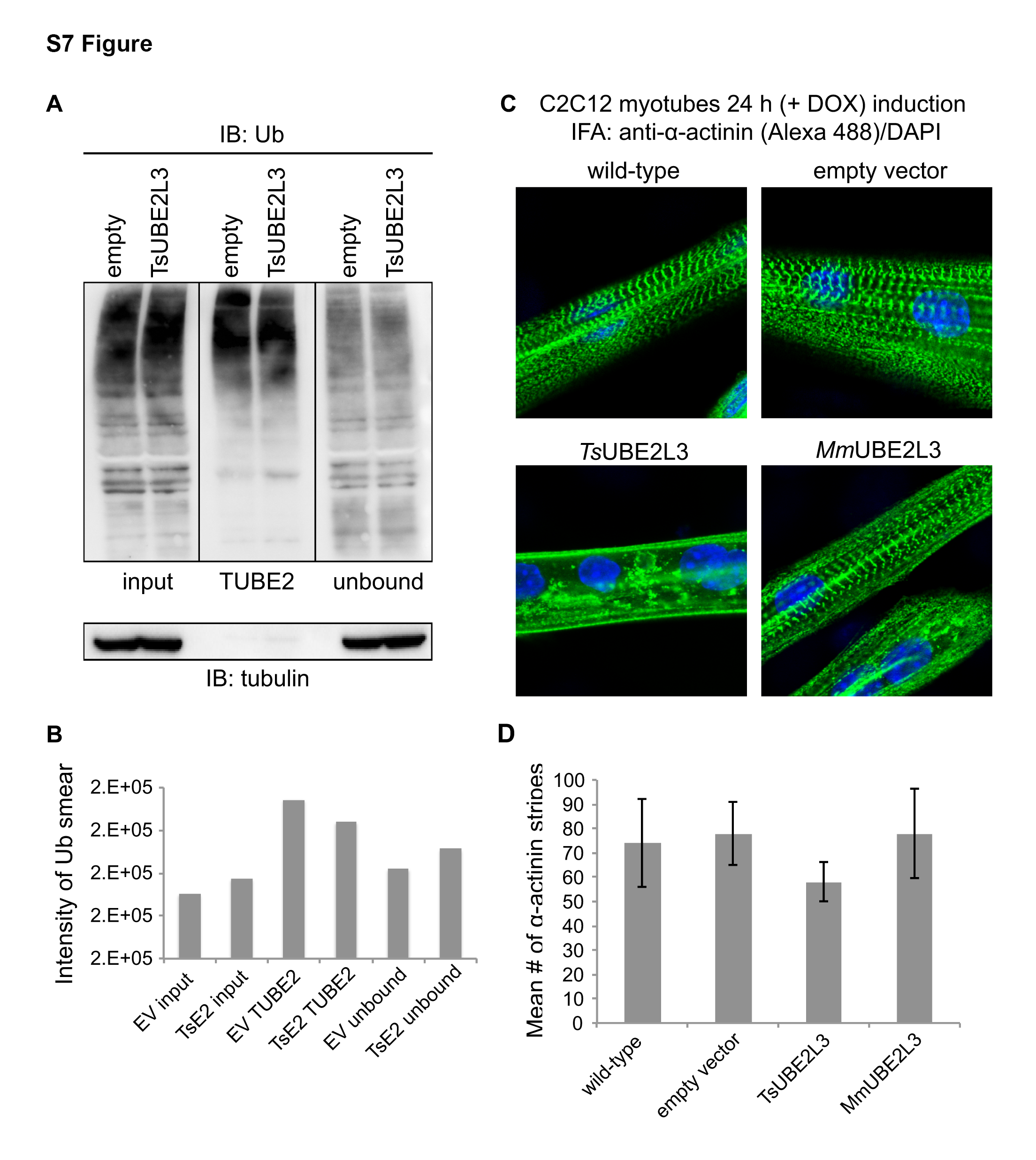

Supplement: S7 Fig — A. TUBE2 IP from transgenic myotube cell lines. Anti-Ub immuno-blot showing normalized lysates of empty vector and TsUBE2L3 C2C12 myotube cells (input), results of tandem ubiquitin binging entity pull-down (TUBE2), and unbound protein. An anti-vinculin immuno-blot of the same samples was included as a loading control. B. Quantification of Ub immuno-blot. ImageJ was used to analyze the intensity (raw pixel area) of each smear from the immuno-blot shown in A. C. IFA of transgenic myotube cell lines. IFA of anti-α-actinin/Alexa488 (with DAPI-stained nuclei) showing fewer ordered sarcomere A-bands in the TsUBE2L3-HA cells than in the wild-type, empty vector and MmUBE2L3 cells. D. ImageJ quantification (using the “analyze stripes” plugin) of the number of striped α-actinin-positive structures in each cell line. The mean number of stripes calculated from the analysis of 7 images (per cell line) taken over 3 independent experiments is displayed for each cell line, with error bars representing the standard error of the mean. (TIF) [file ppat.1005977.s011.tif]
