# Supplementary material for: Ubiquitin-Dependent Modification of Skeletal Muscle by the Parasitic Nematode, Trichinella spiralis
Source: PLoS Pathog. 2016 Nov 21;12(11):e1005977. doi: 10.1371/journal.ppat.1005977 (PMC5117777; doi:10.1371/journal.ppat.1005977)
Supplement: S1 Text — (DOCX) [file ppat.1005977.s004.docx]

**Supplemental Materials and Methods**

**Parasite isolation, culture and collection of secreted proteins**

This study was approved by the Animal Welfare Ethical Review Board at Imperial College London, and was licensed by and performed under the UK Home Office Animals (Scientific Procedures) Act Personal Project License number 70/8193. *T. spiralis* parasites were maintained in female Sprague-Dawley rats. For re-infections, 3500 muscle larvae were used to infect each rat orally. Parasite isolation was done as described by Arden et al. [(Arden et al. 1997)](https://paperpile.com/c/uotHmL/8zPN). Briefly, muscle larvae were isolated from infected rats by digestion of skeletal muscle with 1% v/v HCL and 1% w/v pepsin in tap water at 37°C for 1.5 hours shaking before being filtered through muslin cloth. Muscle larvae were pelleted by gravity, pellets were washed in sterile PBS and cultured in sterile serum-free RPMI (Gibco) supplemented with 1% w/v glucose, 100 U/ml penicillin, 100 µg/ml gentamycin, 20 U/ml nystatin and 2 mM glutamine at 37ºC and 5% CO2 with a daily change of medium for a maximum of 4 days. Culture supernatants containing secreted proteins (SP) were collected at each media change and filtered through a 0.2 µm pore-size membrane. SP were then dialyzed into 25 mM HEPES buffer pH 7.4 and concentrated using 10 kDa molecular weight cut-off (MWCO) vivaspin columns (Sartorius Stedim). SP protein concentrations were measured using a BCA assay (Pierce). Peptide-*N*-Glycosidase F (PNGaseF, NEB) was used to remove *N*- and *O*-linked glycans from *T. spiralis* SP according to the manufacturer’s instructions.

**LC/MS/MS**

Samples were digested with trypsin using standard protocols and peptides were analyzed on either an Orbitrap XL2 (*T. spiralis* SP) or Elite (co-IP) mass spectrometer equipped with an Agilent 1100 binary pump and a Famos microautosampler and separated using a gradient of 6 to 28% acetonitrile in 0.125% formic acid over 90 min. Peptides were detected in the Orbitrap by means of either a data-dependent top 10 method (*T. spiralis* SP) or a top 20 method (co-IP) with a minimum signal intensity of 500 to be selected for MS2. Each full scan was followed by the selection of the most intense ions, up to 10, for collision-induced dissociation (CID) in the linear ion trap. MS2 spectra were searched using SEQUEST v.28 against a composite database derived from the UniProt *Trichinella* *spiralis* proteome, its reversed complement and known contaminants. Peptide spectral matches were filtered to either a 1% (*T. spiralis* SP) false discovery rate (FDR) or a 1.7% FDR (co-IP) using the target-decoy strategy combined with linear discriminant analysis.

**Immuno-blot analyses**

After SDS-PAGE separation, proteins were transferred onto PVDF membrane and blocked for 1.5 h at room temperature in 5% w/v non-fat milk in PBS-Tween (0.1% v/v - PBST), or in 5% BSA w/v in PBST for streptavidin blots. Membranes were then incubated in antibodies diluted in 2% non-fat milk/PBST, or in 2% BSA w/v in PBST for streptavidin blots. Membranes were washed in PBST before being visualized using enhanced chemiluminescence substrate (PerkinElmer). The ImageJ gel analysis plugin was used to quantify pixel intensities of adjacent lanes of immuno-blots using a standardized selected area.

**RACE-PCR and cloning into mammalian and bacterial expression vectors**

Two RACE kits were used (according to manufacturer’s instructions) to process RNA: The FirstChoice RNA Ligase Mediated Rapid Amplification of cDNA Ends (RLM-RACE) kit (Ambion) and the GeneRacer kit (Invitrogen). Nested PCR was then carried out using gene specific primers (GSRP *Ts*UBE2L3: CCAATTCTGTACGAAGCGAATGTTCC, GSFP *Ts*UBE2L3: ACGGAGCACATTATTGATTCGTTGATC) with the RACE primers supplied by the RACE kits. RACE-PCR amplified products were ligated into pGEMTeasy using TA cloning, sequenced (Beckman Coulter Genomics) and diagnostic restriction digests using BamHI and NotI were carried out. Sequences were verified by alignment with *T. spiralis* annotated genomic DNA (Mitreva et al., 2011). Total *T. spiralis* RNA was used to synthesize cDNA using oligo-dT_(12-18)_ primers (M-MLV Reverse Transcriptase, Life Technologies). *Ts*UBE2L3-HA, *Mm*UBE2L3-HA and eGFP were cloned into pLVX Tight Puro and 6His-*Ts*UBE2L3 into pPET28(a)+ (standard methods).

*Ts*UBE2L3 primers:

*Ts*UBE2L3 forward: CC**GGATCC**atgactgcgagtcgtagacttcaaaaagag

*Ts*UBE2L3 reverse: G**CGCGGCCGCtta**GTCGAGTGCGTAGTCTGGTACGTCaacatcctgacgtttttccgcgtat.

The forward primer contained a 5’ BamHI linker sequence (bold capitals) and the reverse primer contained a 3’ NotI linker sequence (bold capitals) a stop codon (bold) and a hemagglutinin (HA) tag (capitals).

**RNA isolation and cloning**

Pelleted *T. spiralis* L1 larvae were frozen in liquid N_2_ before being shattered in a percussive disruptor. Total RNA was extracted using Trizol reagent (Invitrogen) using the manufacturer’s instructions and reverse transcribed into cDNA using an M-MLV Reverse Transcriptase kit (Promega) according to the manufacturer’s instructions. The RACE-confirmed coding sequence for *Ts*UBE2L3 was amplified by PCR (Supplemental Experimental Procedures) [(Mitreva et al. 2011)](https://paperpile.com/c/uotHmL/napp). The PCR product was cloned via pGEMTeasy (Promega) into pLVX Tight Puro mammalian and pPET28a(+) bacterial expression vectors using standard BamHI/NotI restriction enzyme cloning methods. Plasmids were transformed into *E. coli* XL10-Gold competent cells (Agilent Technologies). Mus musculus UBE2L3-HA was cloned from mouse cDNA prepared from mouse skeletal muscle tissue as described above, and eGFP-HA was cloned from a plasmid kindly donated by Dr Andrew Blagborough (Supplemental Experimental Procedures). 6His-*Hs*ARIH2ΔAri was cloned from a pGEX6P1 *Hs*ARIH2-GST vector using the following primers: fw primer (EcoRI) CCGGAATTCATGTCAGTGGACATGAATAGCCA, rev primer (NotI)  AAGGAAAAAAGCGGCCGCCTACCTCGCCTGGGCTTGTTGG based on the HHARI/ARIH1 Ariadne domain truncation described by Duda et al., 2013 [(Duda et al. 2013)](https://paperpile.com/c/uotHmL/NWVf).

**Recombinant protein expression and purification**

Purified pPET28a(+) 6His-*Ts*UBE2L3 and 6His-*Hs*ARIH2ΔAri plasmids were transformed into *E. coli* BL21-CodonPlus (DE3)-RIL competent cells (Agilent Technologies), cultured and selected using standard methods. A clonal population was then grown overnight in 10 ml Luria-Bertani Broth (LB) kanamycin (30 μg/ml) media before inoculating 1 l LB. For 6His-*Ts*UBE2L3 the following methods were used: at an optical density of 0.6 (600 nm) cultures were induced with 0.25 mM IPTG (Isopropyl β-D-1-thiogalactopyranoside) and grown for a further 4 h at 32°C. Cell pellets were lysed in BugBuster (Novagen), diluted in 1x Ni-NTA native lysis buffer (Qiagen) supplemented with benzonase (25U/ml), lysozyme (1mg/ml), PMSF (1mM), Trypsin inhibitor (0.3 μg/ml) and aprotinin 0.025 TIU/ml. Lysates were cleared by centrifugation for 20 min at 16,000xg at 4°C. Recombinant protein was purified from the soluble fraction using Ni-NTA resin (Qiagen) under native conditions according to the manufacturer’s instructions. For 6His-*Hs*ARIH2ΔAri the following methods were used: At an optical density of 1.5 (600 nm) cultures were induced with 1 mM IPTG and grown for a further 16 h at 20°C. Cell pellets were lysed in 1% Triton X-100 in native lysis buffer (Qiagen) using the Constant Cell Disruption System and inclusion bodies were pelleted by centrifugation for 1 h at 40,000xg at 4°C. Subsequently, pellets were resuspended and washed in 2% Triton X-100 buffer, 1M NaCl, native lysis buffer and finally inclusion bodies were lysed overnight at 4°C in 8 M Urea lysis buffer (Qiagen). Lysates were cleared by centrifugation for 20 min at 16,000xg at 4°C. 6His-*Hs*ARIH2ΔAri was then gradually dialyzed using Visking Dialysis Membrane (Medicell) out of denaturing buffers into native protein buffer (50 mM Tris-HCl pH 7.4, 150 mM NaCl) supplemented either with 4, 2 and 1 M Urea at 4°C for 2 h and finally overnight without Urea. Protein concentrations were measured using a BCA assay.

***Ts*UBE2L3 depletion and Ub conjugation assays**

Protein G Dynabead (Life Technologies) precleared *T. spiralis* SP were depleted of *Ts*UBE2L3 by incubating for 1 h at 4°C with Dynabead-bound (Life Technologies)-anti-*Hs*UBE2L3 antibodies in Ub assay buffer (25 mM HEPES pH 7.4 supplemented with 1 mM PMSF, 0.3 μg/ml trypsin inhibitor and 0.025 TIU/ml aprotinin). For negative controls, SP were incubated with protein G Dynabead-bound BSA and anti-tubulin. Dynabead resin was removed from samples and bound proteins were eluted using glycine elution according to the manufacturer’s instructions. Eluted proteins were refolded into Ub assay buffer. For parkin auto-ubiquitination assay reactions were initiated using the Boston Biochem K105 kit according to the manufacturer’s instructions. For all other reactions, the following components (Boston Biochem) were used: UBE1A (E-304, 1 µM), UBE2L3/UbcH7 (E2-640, 1 µM), biotin-Ub:Ub mixture (1:3 ratio, U-570:U100, 250 µM), MgATP (10 mM) and 1x ubiquitin conjugation reaction buffer (B-70). In addition, p-parkin (kindly donated by Wade Harper and Alban Ordureau, Harvard Medical School) [(Ordureau et al. 2014)](https://paperpile.com/c/uotHmL/zLgC) was used at 2 µM, 6His*Ts*UBE2L3 was used at 5 µM, 6His-*Hs*ARIH2ΔAri was used at 5 µM and *T. spiralis* SP at a range of 5-15 µg/µl in Ub assay buffer. Reaction mixtures were initiated by addition of the biotin-Ub:Ub and incubated at 37°C for 1.5 h. Proteins were separated by SDS-PAGE and analyzed by streptavidin-blot.

**Cell culture and differentiation.**

Mouse C2C12 skeletal myoblast cells [(Blau et al. 1985; Yaffe & Saxel 1977)](https://paperpile.com/c/uotHmL/p00z+VY4d) were cultured in growth media (high-glucose DMEM without HEPES or Na+ pyruvate (Gibco) supplemented with 4 mM L-glutamine, 100 U/ml penicillin, 100 μg/ml streptomycin and 20% fetal bovine serum (Gibco)). For experiments using multiple cell lines, myoblasts were seeded at a normalized confluency. Cells were split 1:10 every 2 days using 0.25% v/v trypsin-EDTA and never allowed to grow beyond 60-70% confluency. For differentiation into myotubes, cells were grown to 90% confluency. Media was then switched to differentiation media (high-glucose DMEM without HEPES or Na+ pyruvate supplemented with 2 mM L-glutamine, 100 U/ml penicillin, 100 μg/ml streptomycin and 2% horse serum (Sigma)) and grown for 4 days, changing media daily. HEK 293T cells were cultured in growth media and split 1:20 every 3 days using 0.05% w/v trypsin-EDTA. For experiments cells were harvested by directly lysing in NP-40 lysis buffer (50 mM Tris-HCl pH 7.4, 150 mM NaCl, and 1% NP-40 v/v).

**Lentivirus preparation, transduction of C2C12 cells, generation of cell lines and induction of transgene**

PLVX Tet On (1 ml) and pLVX Tight Puro lentivirus particles were prepared in HEK 293T cells as described by Mostoslavsky et al [(Mostoslavsky et al. 2005)](https://paperpile.com/c/uotHmL/trPV). For the generation of stable C2C12 cell lines, myoblasts were seeded into 6-well plates 24 h prior to transduction to ensure 70% confluency. Equal volumes of Tet On and Tight Puro particles were added with 8 µg/ml Polybrene (Sigma) to each well. Plates were centrifuged for 90 min at 2000xg. Fresh growth media was added 6 h post-transduction, and 48 h post-transduction media was supplemented with geneticin (G418, 500 µg/ml) and puromycin (4 µg/ml) for selection for 5 days. Cells were thereafter maintained in growth media supplemented with 250 µg/ml G418 and 2 µg/ml puromycin. Stable C2C12 cell lines were differentiated into myotubes before transgene induction using 2 µg/ml doxycycline (DOX).

**Immuno-fluorescence analysis (IFA)**

C2C12 myoblasts were seeded into 24-well ibiTreat μ-plates (ibidi) and differentiated as described. After 24 h DOX induction, cells were washed in PBS before processing for IFA using a series of treatments: 20 min fixation at room temperature (RT) in 4% paraformaldehyde in PBS, 3x washes in PBS, 10 min permeabilisation at RT in 0.2% v/v Triton-X and 3x washes in PBS. Samples were blocked overnight at 4°C in blocking buffer (5% BSA w/v, 10% goat serum v/v in PBS). Samples were then incubated in primary antibodies in antibody buffer (3% goat serum v/v, 1% BSA w/v, 0.05% Tween-20 v/v in PBS) for 1 h at RT, followed by secondary antibodies in PBS for 1 h at RT. Samples were washed 3x in PBS before incubation with Hoecht (Invitrogen-1:20,000) for 15 min at RT. Samples were resuspended in PBS and visualized using a Leica SP5 MP/FLIM inverted confocal microscope.

**Immuno-histofluorescence analysis (IHF)**

*T. spiralis* infected rat skeletal muscle tissue was collected 1 month post-infection, dissected and fixed in 10% neutral buffered formalin overnight at 4°C. Tissue was embedded in paraffin and 4 µm sections were cut. Sections were dewaxed in 2x15 min of Histoclear before rinsing twice in ethanol. Antigen retrieval was carried out using trypsin: sections were warmed for 10 min in distilled water at 37°C, 10 min at 37°C 0.1% w/v trypsin (from porcine pancreas) and 0.1% w/v calcium chloride pH 7.8, 10 min in cold running water. Sections were blocked overnight at 4°C in blocking buffer. Samples were then blocked using the Streptavidin/Biotin Blocking Kit (Vector Laboratories) according to the manufacturer’s instructions and incubated in anti-*Ts*UBE2L3 in 3% goat serum v/v, 1% BSA w/v, 0.05% Tween-20 v/v in PBS for 1 h at RT, followed by biotin-anti-rabbit F(ab)2 fragment antibodies (Stratech Scientific) in PBS for 1 h at RT, followed by streptavidin-Dylight 549 (Vector Laboratories) in PBS for 30 min (Supplemental Experimental Procedures for antibody details) and finally Hoecht (1:20,000 for 15 min at RT. Sections were washed 3x in PBS with 0.05% Triton-X-100 between all incubations. Sections were sealed with a glass coverslip and visualized using a Leica SP5 MP/FLIM inverted confocal microscope. For image analysis for quantification of α-actinin-positive Z-band sarcomere stripes, the ImageJ Analyze Stripes algorithm was used. Total numbers of stripes per image were recorded.

**Preparation of myotube lysates, co-immuno-precipitation and TUBE2 pull-downs**

Nuclear and cytosolic extracts were prepared using the CelLyic NuCLEAR Extraction kit (Sigma-Aldrich) according to the manufacturer’s instructions. The cytosolic and nuclear fractions of each sample were pooled, diluted into co-IP buffer (50mM Tris-HCl pH 7.8, 150mM NaCl, 0.5% v/v NP40) and protein concentrations were measured by BCA and normalized. Anti-HA affinity matrix (Roche - 10 µl per 1 ml of lysate) was added and incubated while rocking for 1 h at 4°C. Matrix was pelleted by centrifugation at 4000xg for 5 min and supernatant was removed. Pellets were washed 5x in 1 ml of co-IP buffer supplemented with 0.5 mM PMSF, 1 mM DTT and EDTA-free protease inhibitor cocktail with a final removal of all supernatant using a 26-gauge needle syringe. Pellets were resuspended in 500 µg/ml of HA-peptide (Sigma) in elution buffer (50mM Tris-HCl pH 7.8, 150mM NaCl, 0.5% v/v NP40 and 0.1% w/v SDS) and incubated shaking for 45 min at 50°C. Matrix was pelleted by centrifugation at 4000xg for 5 min and supernatant was collected. Pellets were resuspended in 1 mg/ml of HA-peptide in elution buffer and incubated as above. Matrix was pelleted by centrifugation at 4000xg for 5 min and supernatant was collected. Supernatants were pooled for SDS-PAGE, silver staining and LC/MS/MS analysis. For TUBE2 pull-downs, Agarose-TUBE2 resin (LifeSensors) was used to purify polyubiquitinated proteins according to the manufacturer’s suggested protocol. Elution of polyubiquitinated proteins from the resin was carried out by heating at 96°C in SDS-PAGE loading buffer for 5 minutes.

**Yeast-2-hybrid (Y2H) analysis**

Generation of bait and prey Y2H clones: Y2H assays used the PJ69-4A (bait) and PJ69-4α (prey) yeast strains. Human E3-RING prey clones were constructed as described previously [(Markson et al. 2009; Woodsmith et al. 2012)](https://paperpile.com/c/uotHmL/mSAK+oeCb) using pACTBD/E-B vectors [(Semple et al. 2005)](https://paperpile.com/c/uotHmL/L5M3). The *Ts*UBE2L3 open reading frame was cloned from pGEMTeasy into the bait pGBAE-B Y2H vector through *in vivo* gap repair cloning as previously described [(Ito et al. 1983; Semple et al. 2005)](https://paperpile.com/c/uotHmL/cw8u+L5M3).

Y2H matrix mating assays: The *Ts*UBE2L3 bait clone was mated against arrays of 166 full-length CDS human E3-RING prey clones and 39 prey clones containing the cytoplasmic domains of human transmembrane E3-RING proteins, on YPAD agar plates for 24 h. Yeast colonies were then replicated onto diploid selection media lacking tryptophan and leucine (SD-WL) for a further 48 h. In order to detect yeast expressing positive interaction partners, diploid colonies were replicated onto triple selection media lacking tryptophan, leucine, and either adenine (SD-WLA) or histidine supplemented with 2.5mM 3-Amino-1,2,4-triazole (SD-WLH(AT)). Growth of positive colonies was monitored and scored over a period of 14 days (S6 Figure and S1 Table).

**Cloning for yeast-2-hybrid vector construction**

*Ts*UBE2L3 primer sequences used to amplify ORF for gap repair: forward primer sequence 5’-3’: gaattcacaagtttgtacaaaaaagcaggctggATGACTGCGAGTCGTAGACTTAAAAAG. Reverse primer sequence 5’-3’: CCAGATTACGCTCTCGACCACccagctttcttgtacaaagtggtcgac. Capitals indicate gene specific sequences, lower case indicate vector specific regions.

**Structural Modeling**

Molecular models were generated using Modeller [(Sali & Blundell 1993)](https://paperpile.com/c/uotHmL/pX3x) and MacroModel (Schrodinger, New York, NY). *Ts*UBE2L3 and *Hs*UBE2L3 were modeled using an ensemble of available X-ray crystal structures of UbcH7 in complex with an E3 ligase (69% and 100% sequence identity respectively; PDB ID: 1C4Z [(Huang et al. 1999)](https://paperpile.com/c/uotHmL/zW0J), and UbcH8 (50% and 55% sequence identity respectively; PDB ID: 1WZV). hARIH2 was modeled using the X-ray crystal structures of hARIH1 (99% sequence identity; PDB ID: 4KBL [(Huang et al. 1999)](https://paperpile.com/c/uotHmL/zW0J)). The models were then minimized using the MMF94s forcefield in Sybyl-X 2.1.1 (Certara L.P., St Louis, MO), with the final structure having more than 95% of residues in the allowed region of a Ramachandran plot. Following previous approaches [(Albiston et al. 2008; Ascher et al. 2011; Pires et al. 2016)](https://paperpile.com/c/uotHmL/yobf+CgQQ+4Pfe), zinc ions were manually added to the model of *Hs*ARIH2 after comparison with the zinc-bound hARIH1 structure indicated the conformation of residues in the zinc-binding motifs were identical in the two proteins. The models of *Ts*UBE2L3 and *Hs*UBE2L3 bound to Ubiquitin and the RING1 domain of hARIH2 were built using PIPER (Schrodinger, New York, NY), with the available X-ray crystal structures of RING E3’s complexes with E2 (PDB ID: 3HCT, 4AP4 and 4AUQ [(Dou et al. 2012; Plechanovova et al. 2012; Yin et al. 2009)](https://paperpile.com/c/uotHmL/lnVw+mXE5+bIJk) used to guide protein docking. The models of the complex were consistent with those presented by [(Huang et al. 1999)](https://paperpile.com/c/uotHmL/zW0J) and were minimized using the MMF94s forcefield in Sybyl-X 2.1.1 as described above. The quality of all the models were confirmed with Verify3D [(Eisenberg et al. 1997)](https://paperpile.com/c/uotHmL/rVaK) (data not shown). The structural consequences of the differences in interfacial residues were analyzed to assess the structural importance of the residues [(Jubb et al. 2015; Pires et al. 2014b; Pires et al. 2014a)](https://paperpile.com/c/uotHmL/fOTN+Bl9z+EiRC). Model structures were examined using Pymol.

**List of antibodies and probes**

HRP-conjugated streptavidin (Pirece, 1:20,000), HRP conjugated anti-HA (3F10 Roche 12158167001, 1:1000), anti-*Hs*UBE2L3 (LSBio LS-C333117, 1:500), anti-myogenin (Merck Millipore, 1:1000 MAB3876), anti-MHC (Merck Millipore 05-716, 1:1000), anti anti-fast skeletal myosin (myosin II – Abcam ab7784, 1:1000), biotin-anti-rabbit F(ab) fragment (Stratech Scientific, 1:500), streptavidin-conjugated Dylight 549 (Vector Labs, 1:1000), anti-tubulin (Sigma, T9026, 1:1000) anti-vinculin (Abcam ab11194, 1:3000). HRP conjugated anti-rabbit (Amersham NA934, 1:50,000) and anti-mouse (Pierce 31430, 1:10,000). Affinity purified anti-*Ts*UBE2L3 (rabbit – custom-made by Davids Biotechnology) was used at 1:500 for IB and 1:50 for IHF. Anti-sarcomeric α-actinin antibody [EA-53] (Abcam ab9465) was used at 1:50 for IFA.

**UbiScan-LC/MS/MS**

As described by [(Guo et al. 2014; Lee et al. 2011; Rush et al. 2005)](https://paperpile.com/c/uotHmL/7DGA+OCl0+1fId) Ubiquitinated peptide enrichment was performed using the Ubiquitin Branch Motif Antibody (K-ε-GG) #3925 (Cell Signaling Technology). Cell pellets were trypsin-digested, and separated by solid phase extraction using Sep-Pak C18 cartridges. Peptides were lyophilized, re-dissolved and ubiquitin peptides were isolated using K-ε-GG antibody-immobilized to protein A agarose. Resin was washed and ubiquitinated peptides were eluted from antibody resin into 0.15% TFA, desalted with C18 stage tips and loaded directly onto a 10-cm × 75-μm PicoFrit capillary column packed with Magic C18 AQ reversed phase resin for LC/MS/MS analysis. The column was developed with a 90-minute linear gradient of acetonitrile in 0.125% formic acid delivered at 280 nL/min. Tandem mass spectra were collected with an LTQ-Orbitrap-VELOS mass spectrometer (ESI-CID). MS Parameter Settings were as follows: MS Run Time 120 min, MS1 Scan Range (300.0-1500.00), Top 20 MS/MS (Min Signal 500, Isolation Width 2.0, Normalized Coll. Energy 35.0, Activation-Q 0.250, Activation Time 20.0, Lock Mass 371.101237, Charge State Rejection Enabled, Charge State 1+ Rejected, Dynamic Exclusion Enabled, Repeat Count 1, Repeat Duration 35.0, Exclusion List Size 500, Exclusion Duration 40.0, Exclusion Mass Width Relative to Mass, Exclusion Mass Width 10ppm).

**Supplemental Materials and Methods References**

[Albiston, A.L. et al., 2008. Identification and characterization of a new cognitive enhancer based on inhibition of insulin-regulated aminopeptidase. *FASEB journal: official publication of the Federation of American Societies for Experimental Biology*, 22(12), pp.4209–4217.](http://paperpile.com/b/uotHmL/yobf)

[Arden, S.R. et al., 1997. Identification of serine/threonine protein kinases secreted by Trichinella spiralis infective larvae. *Molecular and biochemical parasitology*, 90(1), pp.111–119.](http://paperpile.com/b/uotHmL/8zPN)

[Ascher, D.B. et al., 2011. Regulation of insulin-regulated membrane aminopeptidase activity by its C-terminal domain. *Biochemistry*, 50(13), pp.2611–2622.](http://paperpile.com/b/uotHmL/CgQQ)

[Blau, H.M. et al., 1985. Plasticity of the differentiated state. *Science*, 230(4727), pp.758–766.](http://paperpile.com/b/uotHmL/p00z)

[Dou, H. et al., 2012. BIRC7-E2 ubiquitin conjugate structure reveals the mechanism of ubiquitin transfer by a RING dimer. *Nature structural & molecular biology*, 19(9), pp.876–883.](http://paperpile.com/b/uotHmL/lnVw)

[Duda, D.M. et al., 2013. Structure of HHARI, a RING-IBR-RING ubiquitin ligase: autoinhibition of an Ariadne-family E3 and insights into ligation mechanism. *Structure* , 21(6), pp.1030–1041.](http://paperpile.com/b/uotHmL/NWVf)

[Eisenberg, D., Lüthy, R. & Bowie, J.U., 1997. VERIFY3D: assessment of protein models with three-dimensional profiles. *Methods in enzymology*, 277, pp.396–404.](http://paperpile.com/b/uotHmL/rVaK)

[Guo, A. et al., 2014. Immunoaffinity enrichment and mass spectrometry analysis of protein methylation. *Molecular & cellular proteomics: MCP*, 13(1), pp.372–387.](http://paperpile.com/b/uotHmL/7DGA)

[Huang, L. et al., 1999. Structure of an E6AP-UbcH7 complex: insights into ubiquitination by the E2-E3 enzyme cascade. *Science*, 286(5443), pp.1321–1326.](http://paperpile.com/b/uotHmL/zW0J)

[Ito, H. et al., 1983. Transformation of intact yeast cells treated with alkali cations. *Journal of bacteriology*, 153(1), pp.163–168.](http://paperpile.com/b/uotHmL/cw8u)

[Jubb, H., Blundell, T.L. & Ascher, D.B., 2015. Flexibility and small pockets at protein–protein interfaces: New insights into druggability. *Progress in biophysics and molecular biology*, 119(1), pp.2–9.](http://paperpile.com/b/uotHmL/fOTN)

[Lee, K.A. et al., 2011. Ubiquitin ligase substrate identification through quantitative proteomics at both the protein and peptide levels. *The Journal of biological chemistry*, 286(48), pp.41530–41538.](http://paperpile.com/b/uotHmL/OCl0)

[Markson, G. et al., 2009. Analysis of the human E2 ubiquitin conjugating enzyme protein interaction network. *Genome research*, 19(10), pp.1905–1911.](http://paperpile.com/b/uotHmL/mSAK)

[Mitreva, M. et al., 2011. The draft genome of the parasitic nematode Trichinella spiralis. *Nature genetics*, 43(3), pp.228–235.](http://paperpile.com/b/uotHmL/napp)

[Mostoslavsky, G. et al., 2005. Efficiency of transduction of highly purified murine hematopoietic stem cells by lentiviral and oncoretroviral vectors under conditions of minimal in vitro manipulation. *Molecular therapy: the journal of the American Society of Gene Therapy*, 11(6), pp.932–940.](http://paperpile.com/b/uotHmL/trPV)

[Ordureau, A. et al., 2014. Quantitative proteomics reveal a feedforward mechanism for mitochondrial PARKIN translocation and ubiquitin chain synthesis. *Molecular cell*, 56(3), pp.360–375.](http://paperpile.com/b/uotHmL/zLgC)

[Pires, D.E.V. et al., 2016. In silico functional dissection of saturation mutagenesis: Interpreting the relationship between phenotypes and changes in protein stability, interactions and activity. *Scientific reports*, 6, p.19848.](http://paperpile.com/b/uotHmL/4Pfe)

[Pires, D.E.V., Ascher, D.B. & Blundell, T.L., 2014a. DUET: a server for predicting effects of mutations on protein stability using an integrated computational approach. *Nucleic acids research*, 42(Web Server issue), pp.W314–9.](http://paperpile.com/b/uotHmL/EiRC)

[Pires, D.E.V., Ascher, D.B. & Blundell, T.L., 2014b. mCSM: predicting the effects of mutations in proteins using graph-based signatures. *Bioinformatics* , 30(3), pp.335–342.](http://paperpile.com/b/uotHmL/Bl9z)

[Plechanovova, A. et al., 2012. Rnf4 - ubch5a - ubiquitin heterotrimeric complex. Available at:](http://paperpile.com/b/uotHmL/mXE5) <http://dx.doi.org/10.2210/pdb4ap4/pdb>[.](http://paperpile.com/b/uotHmL/mXE5)

[Rush, J. et al., 2005. Immunoaffinity profiling of tyrosine phosphorylation in cancer cells. *Nature biotechnology*, 23(1), pp.94–101.](http://paperpile.com/b/uotHmL/1fId)

[Sali, A. & Blundell, T.L., 1993. Comparative protein modelling by satisfaction of spatial restraints. *Journal of molecular biology*, 234(3), pp.779–815.](http://paperpile.com/b/uotHmL/pX3x)

[Semple, J.I. et al., 2005. Two-hybrid reporter vectors for gap repair cloning. *BioTechniques*, 38(6), pp.927–934.](http://paperpile.com/b/uotHmL/L5M3)

[Woodsmith, J., Jenn, R.C. & Sanderson, C.M., 2012. Systematic analysis of dimeric E3-RING interactions reveals increased combinatorial complexity in human ubiquitination networks. *Molecular & cellular proteomics: MCP*, 11(7), p.M111.016162.](http://paperpile.com/b/uotHmL/oeCb)

[Yaffe, D. & Saxel, O., 1977. Serial passaging and differentiation of myogenic cells isolated from dystrophic mouse muscle. *Nature*, 270(5639), pp.725–727.](http://paperpile.com/b/uotHmL/VY4d)

[Yin, Q. et al., 2009. E2 interaction and dimerization in the crystal structure of TRAF6. *Nature structural & molecular biology*, 16(6), pp.658–666.](http://paperpile.com/b/uotHmL/bIJk)
